# Supplementary material for: Bidirectional contact tracing could dramatically improve COVID-19 control
Source: Nat Commun. 2021 Jan 11;12:232. doi: 10.1038/s41467-020-20325-7 (PMC7801385; doi:10.1038/s41467-020-20325-7)
Supplement: Supplementary file 2 — Supplementary Information [file 41467_2020_20325_MOESM2_ESM.pdf]

Bidirectional contact tracing could dramatically improve COVID-19 control

William J. Bradshaw[1], Ethan C. Alley[2,3], Jonathan H. Huggins[4], Alun L. Lloyd[5], and Kevin M. Esvelt[2,*]

[1]Max Planck Institute for Biology of Ageing, Joseph-Stelzmann-Str. 296, 50937 Cologne, Germany

[2]Media Laboratory, Massachusetts Institute of Technology, Cambridge, MA 02139, USA

[3]Alt. Technology Labs, Berkeley, CA 94702, USA

[4]Department of Mathematics & Statistics, Boston University, Boston, MA 02215, USA

[5]Biomathematics Graduate Program and Department of Mathematics, North Carolina State University, Raleigh, NC 27695, USA

* For correspondence: esvelt@mit.edu

Supplementary Information

Supplementary Methods

Structure of the model - Infection dynamics

A new case is infected at some **exposure time**, equal to zero if the case is an index case and otherwise drawn from the **generation time distribution** of its parent case (see below). If not asymptomatic, the case develops symptoms at some **onset time** drawn from an **incubation time distribution**. Asymptomatic cases do not develop symptoms, but are still assigned an onset time for the purpose of determining their generation-time distribution (see below).

The number of child cases infected by the case is drawn from a negative binomial distribution, with mean equal to the appropriate reproduction number (see below) and heterogeneity determined by the overdispersion parameter k. The exposure times of these child cases are drawn from a skewed-normal **generation time distribution** centered on the symptom onset of their parent[20], with an SD parameter (ω) of 2 and a skew parameter (α) chosen to give a pre-specified probability of pre-symptomatic transmission (for a symptomatic parent) (Supplementary Table 1 & Supplementary Fig. 33). The generation time distribution for an asymptomatic parent is centered on its "effective" onset time (see above). The shape of the generation-time distribution is the same for all cases.

This implementation of the generation time distribution has the advantage of allowing the proportion of pre-symptomatic transmission to be directly specified. However, as a case's generation time value is independent of its incubation time, it is possible for the time between successive exposure times in a chain of transmission to be unrealistically small or even zero. To investigate this potential problem, we simulated draws from the incubation and generation time distributions for the median, optimistic and pessimistic scenarios (Supplementary Table 1) as well as a range of other α values (Supplementary Fig. 33). However, even in the pessimistic scenario, fewer than 2% of cases had a generation time of zero and fewer than 3.5% had a generation time of less than 0.5, levels which we concluded would not have a significant effect on our results.

The expected number of children produced by a case depends on its symptomatic status, and is determined by the overall R_0 value, the proportion of asymptomatic carriers p_{asym}, and the relative infectiousness x_{asym} of asymptomatic carriers (expressed as a fraction of R_0). Given a reproduction number for asymptomatics of $R_{asym} = R_0 \cdot x_{asym}$, the reproduction number of symptomatic cases that produces the desired overall R_0 is given by $R_{sym} = R_{asym} \cdot \frac{p_{asym}}{1-p_{asym}}$.

Structure of the model - Infection control

Once symptoms develop, a case is **identified** by public health authorities with probability p_{isol}, with the delay from onset to identification drawn from a **delay distribution**. Identified cases are instructed to isolate, and each case complies with that order with probability p_{comply}. Cases that comply with isolation generate no further child cases after their time of identification. Asymptomatic cases cannot be identified from symptoms, but may be identified via contact tracing from other cases (see below); once identified, they are instructed to isolate as above. Tracing can also cause symptomatic cases to be isolated earlier than they would be from symptoms alone.

An identified case is **tested**, which takes time drawn from a test time distribution and returns a positive result with probability equal to the sensitivity of the test (since the model does not consider uninfected individuals, the specificity of the test is also not considered). For asymptomatic cases, or symptomatic cases identified prior to symptom onset, a positive test result is required to initiate contact tracing; symptomatic cases that have already developed symptoms can either be traced immediately upon identification, or require a positive test result prior to tracing, depending on model settings.

Whether before or after a test result is obtained, the contacts of an identified case can also be **traced**. Tracing can only proceed outward from a case if they share their contact history, either via a contact-tracing app or with a

manual contact tracer (see below). Tracing can identify the children of the traced case (forward tracing), and may also be able to identify its parent (reverse/backward tracing), depending on model settings. The speed and success probability of tracing depend upon whether tracing is conducted digitally or manually, which in turn depends on several factors:

- If the contact between the trace originator and the tracee occurred environmentally (determined with probability p_{env}), tracing cannot take place.
- If transmission was not environmental, the contact can be traced digitally if:
  - Both the trace originator and the tracee possess chirp-enabled smartphones (determined independently for each individual case with probability $p_{smartphone}$);
  - The trace originator shares their data with the tracing app (determined independently for each individual case with probability $p_{share\_digital}$);
  - The time of between contact (equal to the exposure time of the child case, i.e. of the trace in forward tracing and the trace initiator in reverse tracing) and trace initiation is less than the **data-retention window** of the digital tracing system;
  - The contact between the two cases was recorded by the tracing app of the trace originator (determined independently for each individual case with probability $p_{trace\_digital}$).
- If any of the above conditions are not met, but transmission was not environmental, the contact might still be traced manually if:
  - The trace originator shares their contact history with a manual contact tracer (determined independently for each individual case with probability p_{share_manual});
  - The time between contact (as above) and the identification time or symptom onset of the trace initiator (whichever came first) is less than the **contact-tracing window** of the manual tracing system;
  - The tracee is successfully traced by the contact tracer (determined independently for each individual case with probability p_{trace_manual}).
- If neither digital nor manual tracing succeeds, then the trace fails and the tracee is not traced.

Cases that are successfully traced are identified at a time equal to the **trace initiation time** of the trace originator plus a delay time drawn from the appropriate **trace delay distribution** (which will differ between digital and manual tracing). Cases identified through tracing can then be isolated, tested, and traced as described above. If a

case is isolated through tracing earlier than they would have been otherwise, child cases whose exposure time would be later than their parent's new isolation time are eliminated, as are their descendents.

Run initiation and termination

A simulation of an outbreak under the branching-process model is initialised with a given number of index cases (by default 20, in order to reduce the probability of stochastic elimination) and proceeds generation by generation until either no further child cases are generated (extinction) or the run exceeds one of:

1. A **cumulative case limit** of 10,000 cases, reached if the total number of cases ever exceeded that number, or
2. A **time limit** of 52 weeks, reached if the latest exposure time across all cases ever exceeded that number.

In practice, virtually all runs either went extinct or reached the cumulative case limit; across all scenarios tested for all datasets used in Figures 2-4, the overall percentage of runs that terminated as a result of exceeding the time limit was less than 0.02%, and the highest percentage observed for any single scenario was 1.3%. The cumulative case limit, meanwhile, was selected to minimise the chance of a run that would otherwise go extinct being terminated prematurely while preserving computational tractability; in test runs with a cumulative case limit of 100,000 cases, fewer than 2% of extinct runs in any scenario had a cumulative case count of over 10,000.

A terminated run was deemed "controlled" if it reached extinction, and uncontrolled otherwise. The control rate for a scenario was computed as the proportion of runs for that scenario that were controlled. 95% credible intervals on the control rate were computed by beta-binomial conjugacy under a $Beta(1, 1)$ uniform prior, as the 2.5th and 97.5th percentiles of the beta distribution $Beta(1+k,\ 1+n-k)$, where n is the total number of runs for that scenario and k is the number of controlled runs. Effective reproduction numbers were computed as the mean number of child cases produced across all cases in a run, averaged across all runs in the scenario. For main figures, 1000 runs were performed per scenario; for figures, either 500 or 1000 runs were performed, as specified in the figure captions.

Supplementary tables

Table S1: Parameters of the branching-process model.

**Parameter**	**Value**			**Sources and Notes**
	Median	Optimistic	Pessimistic	
% asymptomatic carriers	45%	40%	55%	[8–10,21]
Relative infectiousness of asymptomatic carriers	50%	45%	60%	Informed by viral loads and tracing results described in [5,9,22–25]
% environmental transmission	10%	5%	15%	[17,26]
Proportion of pre-symptomatic transmission	48%	38%	53%	Informed by viral load measurements, tracing results, and negative serial intervals described in [5,24,25,27–32]
Generation time skew parameter (α)	0.064	0.397	-0.095	Correspond to pre-symptomatic transmission rates specified above.
% of symptomatic cases identified without tracing	50%			[4]
% of cases with chirping smartphones (high-uptake case)	53% (low-uptake) / 80% (high-uptake)			Survey data on phone ownership and attitudes to exposure notification[33–35]
% of cases who comply with isolation	90%			Assumed
% of cases with smartphones who upload data when diagnosed	90%			Assumed
% of cases who share data with manual contact tracers	98%			Assumed
Test sensitivity	70%			[36,37]
R_0 (default)	2.5			Most estimates cluster between 2.0 and 3.0: [9,17,24,38–40]
Overdispersion	0.11			[11]
Number of initial cases	20			Assumed
Data retention window for digital tracing (days)	14 days			[43]
Incubation period	5.5 ± 2.1 days (lognormal distribution)			[41]
Delay from onset to isolation	3.8 ± 2.4 days (Weibull distribution)			[20]
Delay for testing	1 ± 0.3 days (gamma distribution)			Assumed
Delay for manual tracing	1.5 ± 4.8 days (lognormal distribution); median 0.5 days			Previous reports suggest most contacts can be traced within one day, but some take much longer [42]
Delay for digital tracing	0 days			Assumed

Supplementary figures

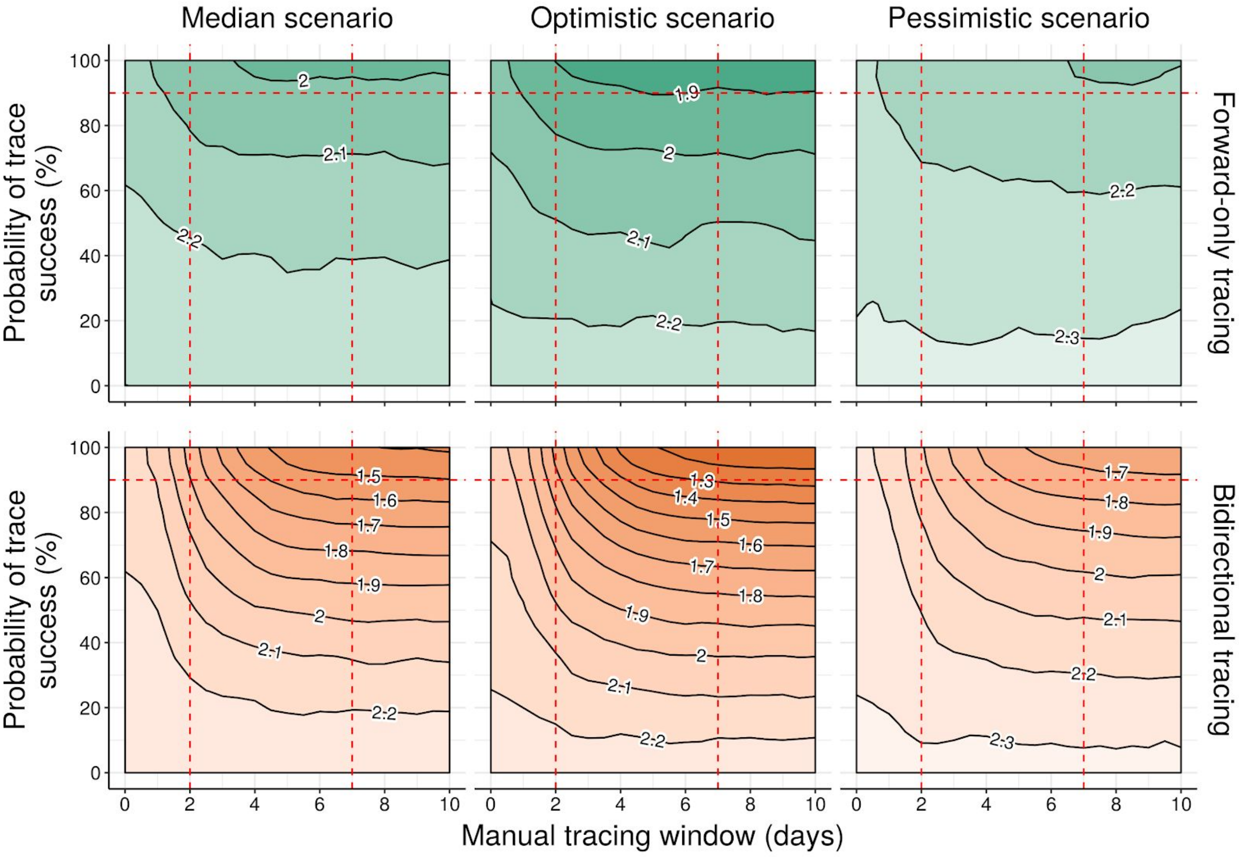

Supplementary Figure 1: Effect of contact-tracing window size on performance of manual tracing. Neighbour-averaged contour plots showing the average effective reproduction number achieved across 1000 runs per condition, under varying manual tracing strategies (panels), tracing window sizes (x-axis), and rates of trace success (y-axis), and assuming otherwise median disease parameters (Supplementary Table 1). The manual tracing window denotes the length of time prior to symptom onset (for symptomatic cases) or case identification (for presymptomatic and asymptomatic cases) in which contacts can be identified. Dashed red lines indicate values used in main-text figures. Note that "probability of trace success" excludes traces blocked by environmental transmission or network fragmentation.

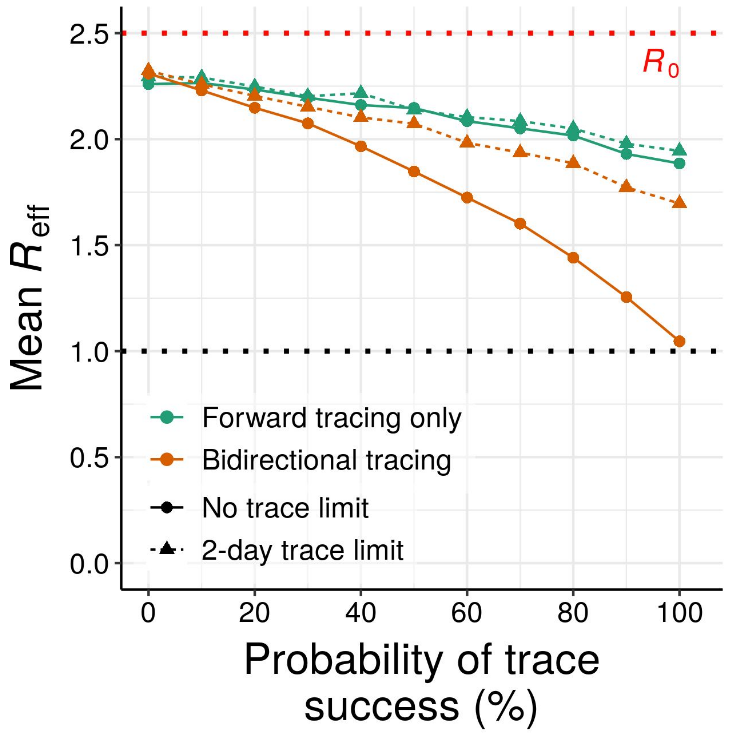

Supplementary Figure 2. Performance of digital exposure notification under universal uptake. Mean R_{eff} achieved (across 1000 runs per condition) by digital exposure notification in the absence of manual tracing, assuming universal smartphone coverage and data sharing, a 14-day data-retention window, and otherwise median disease parameters (Supplementary Table 1). "Probability of trace success" refers to trace attempts that are not otherwise blocked by environmental transmission or fragmentation of the digital network.

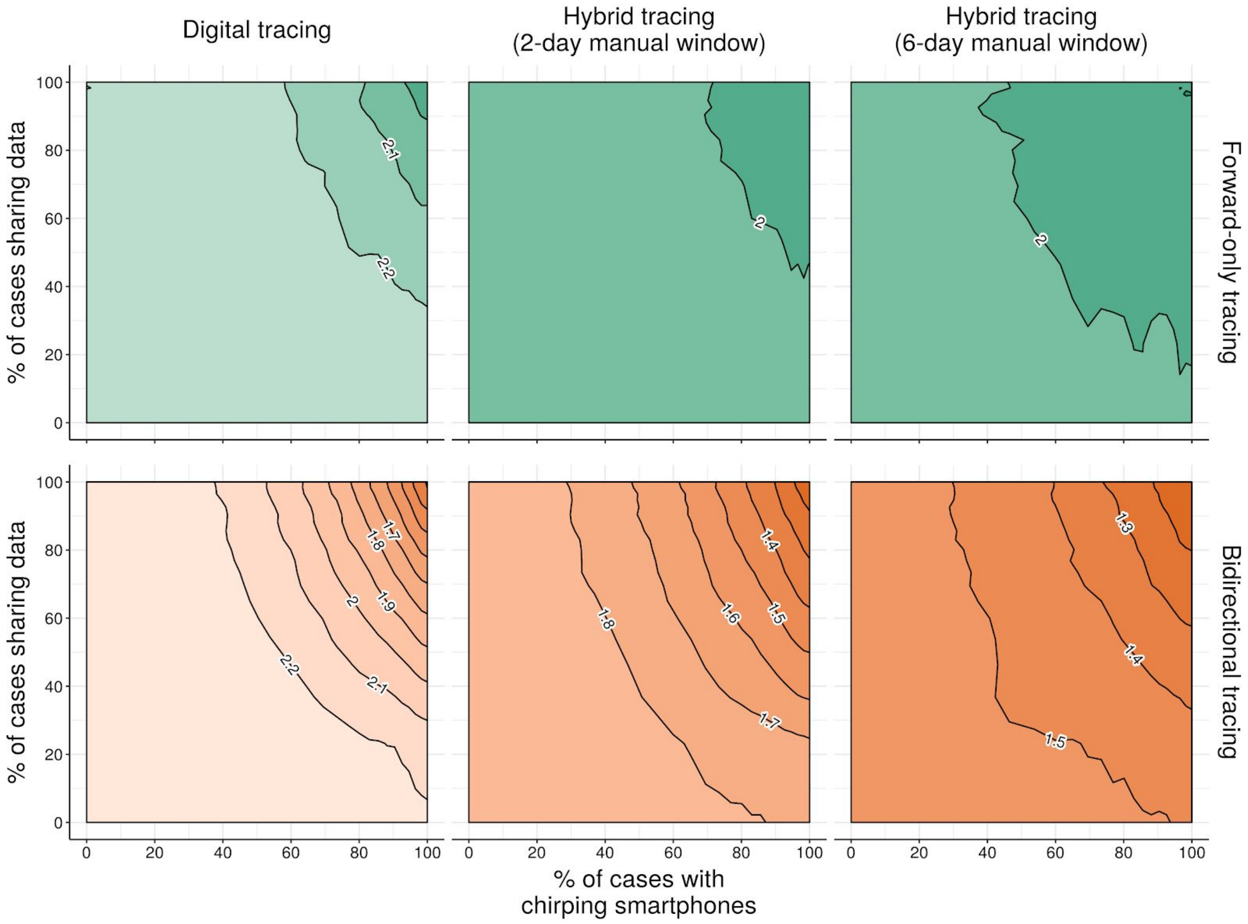

Supplementary Figure 3: Effect of network fragmentation on tracing performance. Neighbour-averaged contour plots of the mean effective reproduction number achieved across 1000 runs per condition, under varying tracing strategies (panels), chirping coverage levels (x-axis) and digital data sharing (y-axis), and assuming 90% probability of trace success and otherwise median disease parameters (Supplementary Table 1). The manual tracing window denotes the length of time prior to symptom onset (for symptomatic cases) or case identification (for presymptomatic and asymptomatic cases) in which contacts can be identified. Digital tracing was assumed to employ a data-retention window of 14 days in all scenarios (Methods). Note that "probability of trace success" excludes traces blocked by environmental transmission or network fragmentation.

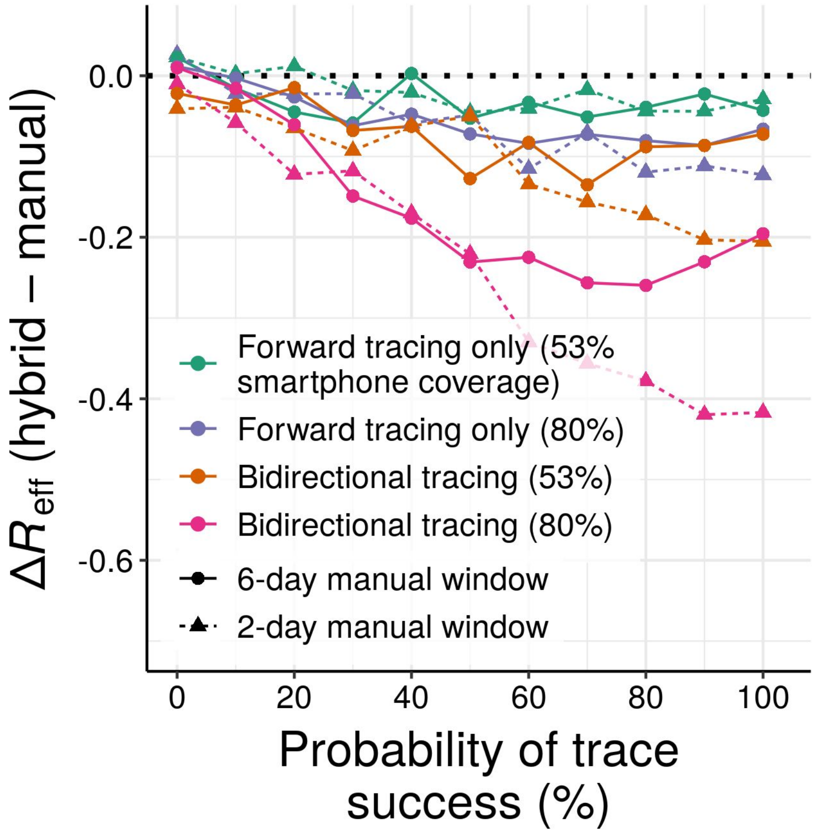

Supplementary Figure 4. Outperformance of hybrid over manual tracing.. Difference between the mean R_{eff} achieved (across 1000 runs per condition) under hybrid (manual + digital) tracing and that achieved by manual tracing under similar conditions, assuming a 14-day data-retention window for digital tracing and median disease parameters (Supplementary Table 1). "Probability of trace success" refers to trace attempts that are not otherwise blocked by environmental transmission or fragmentation of the digital network.

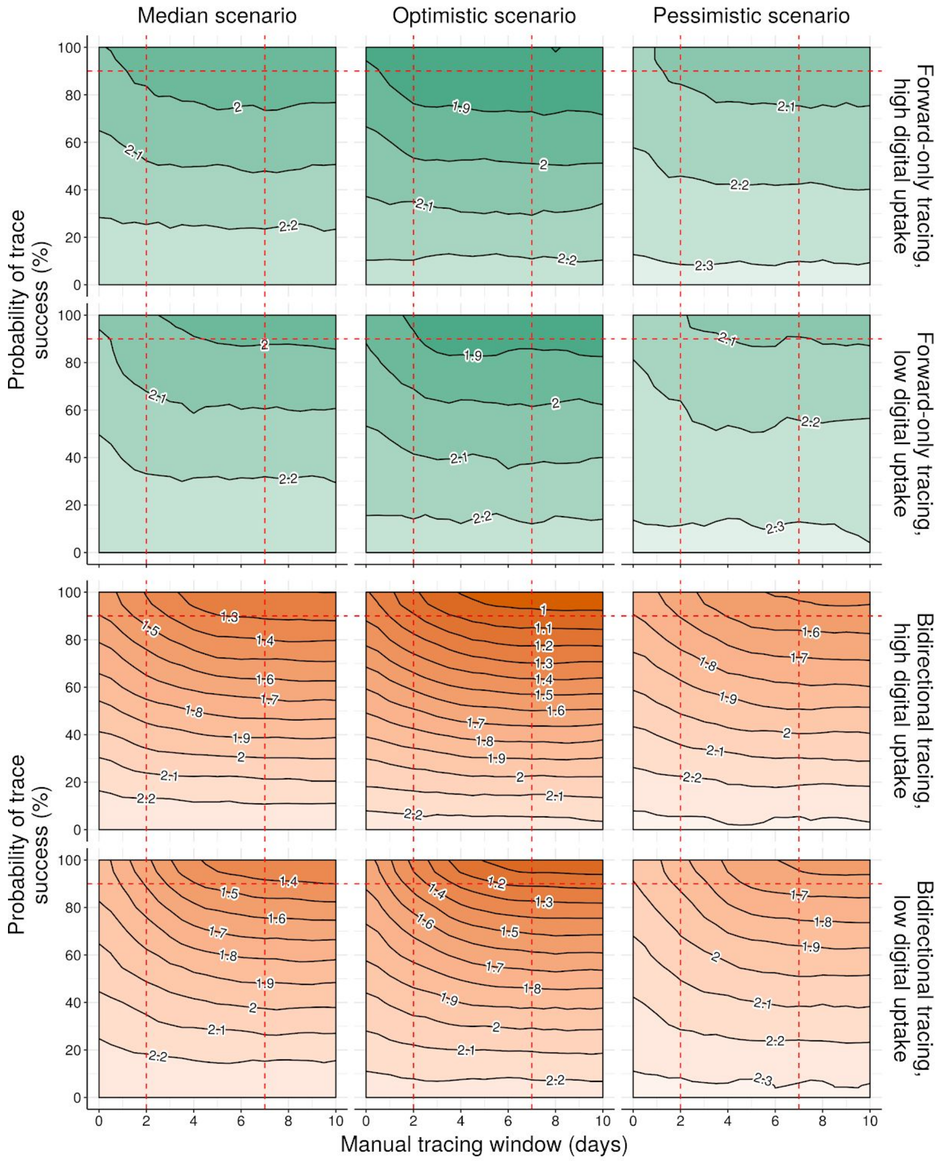

Supplementary Figure 5: Effect of contact-tracing window size on performance of hybrid tracing. Neighbour-averaged contour plots showing the mean effective reproduction number achieved across 1000 runs per condition, under varying hybrid tracing strategies (panels), tracing window sizes (x-axis), and rates of trace success (y-axis), and assuming otherwise median disease parameters (Supplementary Table 1). The manual tracing window denotes the length of time prior to symptom onset (for symptomatic cases) or case identification (for presymptomatic and asymptomatic cases) in which contacts can be identified. Digital tracing was assumed to employ a data-retention window of 14 days in all scenarios (Methods). Dashed red lines indicate values used in main-text figures. Note that "probability of trace success" excludes traces blocked by environmental transmission or network fragmentation.

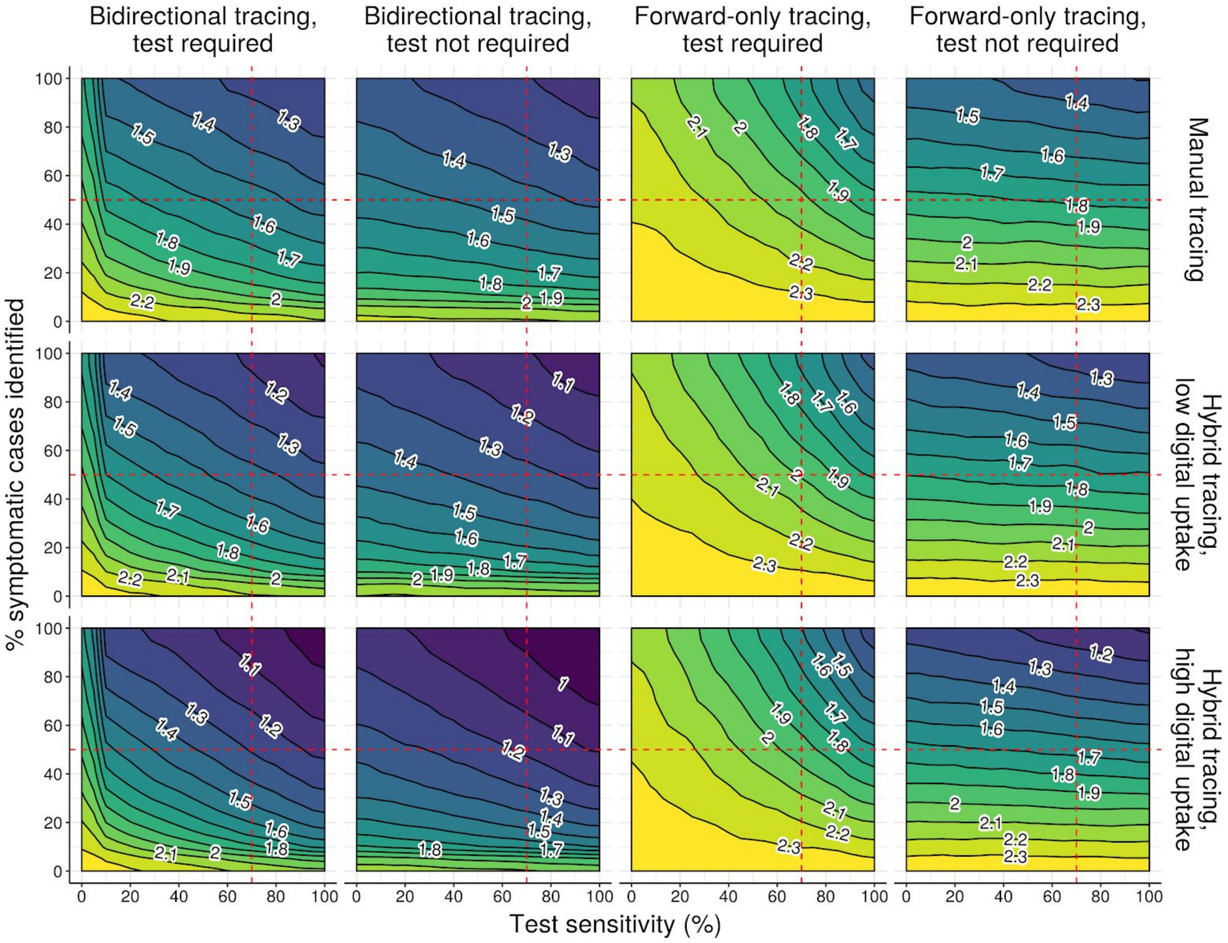

Supplementary Figure 6: Effect of test ascertainment and sensitivity on tracing performance. Neighbour-averaged contour plots of the mean effective reproduction number achieved across 1000 runs per condition, under varying tracing strategies (panels), test sensitivities (x-axis) and percentages of symptomatic cases that can be detected based on symptoms alone (y-axis), and assuming 90% probability of trace success, a 6-day manual tracing window, and otherwise median disease parameters (Supplementary Table 1). "Test required" indicates that a positive test result was required before initiating contact tracing from a symptomatic case, while "test not required" indicates that tracing could be initiated based on symptoms alone. Dashed red lines indicate values used in main-text figures.

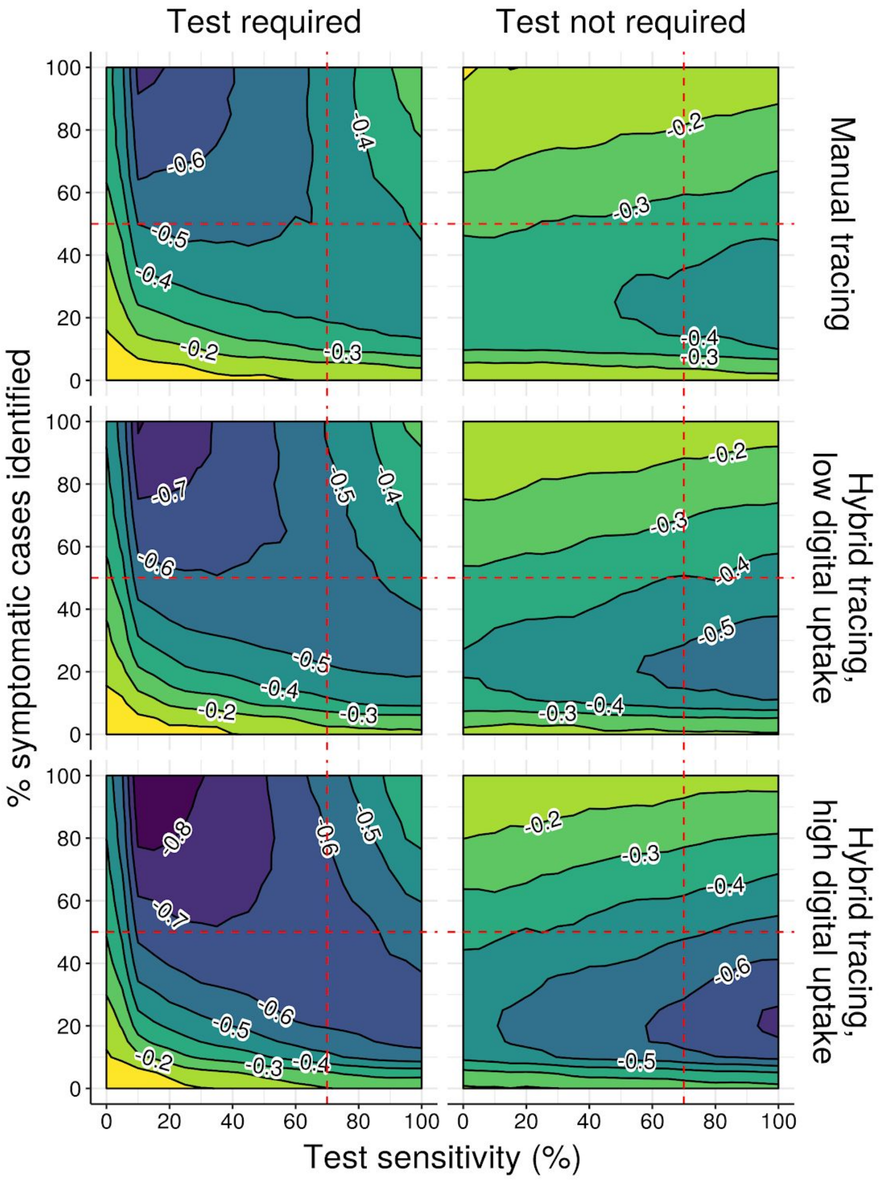

Supplementary Figure 7: Effect of test ascertainment and sensitivity on outperformance of bidirectional tracing. Neighbour-averaged contour plots of the average difference in effective reproduction number achieved by bidirectional relative to forward-only tracing, across 1000 runs per condition, under varying tracing strategies (panels), test sensitivities (x-axis) and percentages of symptomatic cases that can be detected based on symptoms alone (y-axis), and assuming 90% probability of trace success, a 6-day manual tracing window, and otherwise median disease parameters (Supplementary Table 1). "Test required" indicates that a positive test result was required before initiating contact tracing from a symptomatic case, while "test not required" indicates that tracing could be initiated based on symptoms alone. Dashed red lines indicate values used in main-text figures.

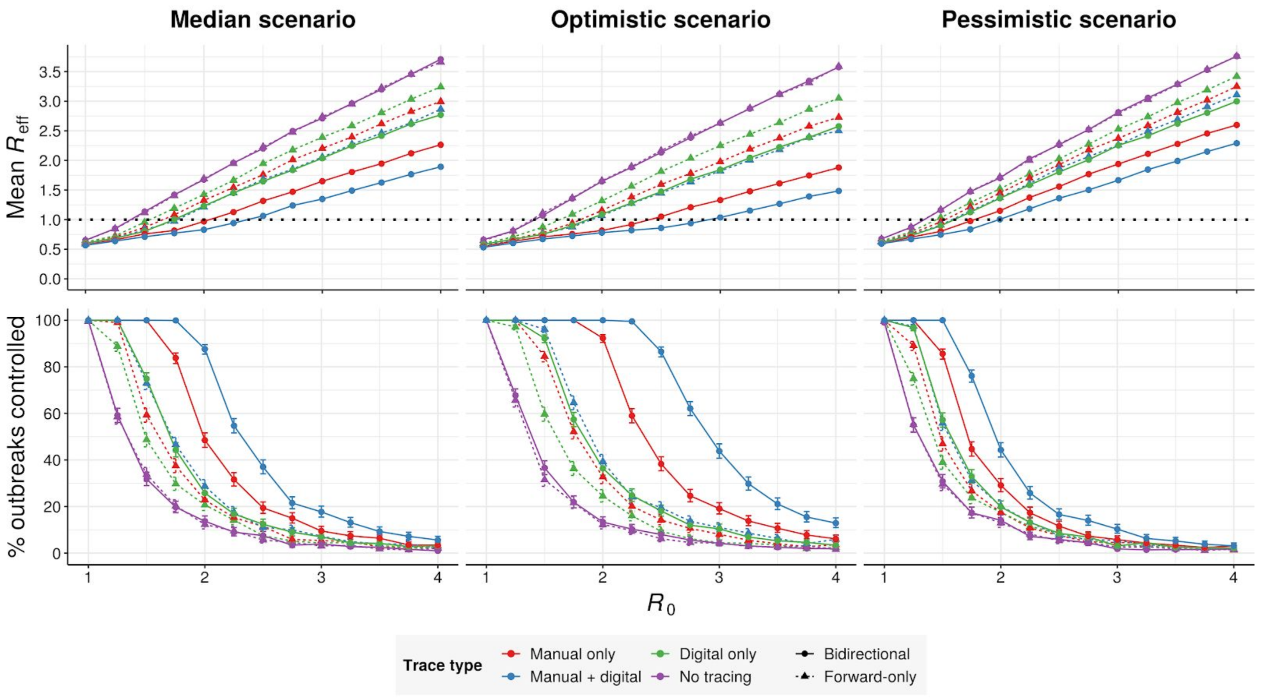

Supplementary Figure 8. Effect of R_0 and disease parameters on performance (high ascertainment). As Fig. 4, but assuming that 90% of symptomatic cases can be detected based on symptoms alone. (top row) Mean R_{eff} achieved and (bottom row) % of outbreaks controlled by different tracing strategies as a function of the basic reproduction number R_0, assuming (left) median, (middle) optimistic or (right) pessimistic disease parameters (Supplementary Table 1). Error bars in the bottom row represent 95% credible intervals across 1000 runs under a uniform beta prior.

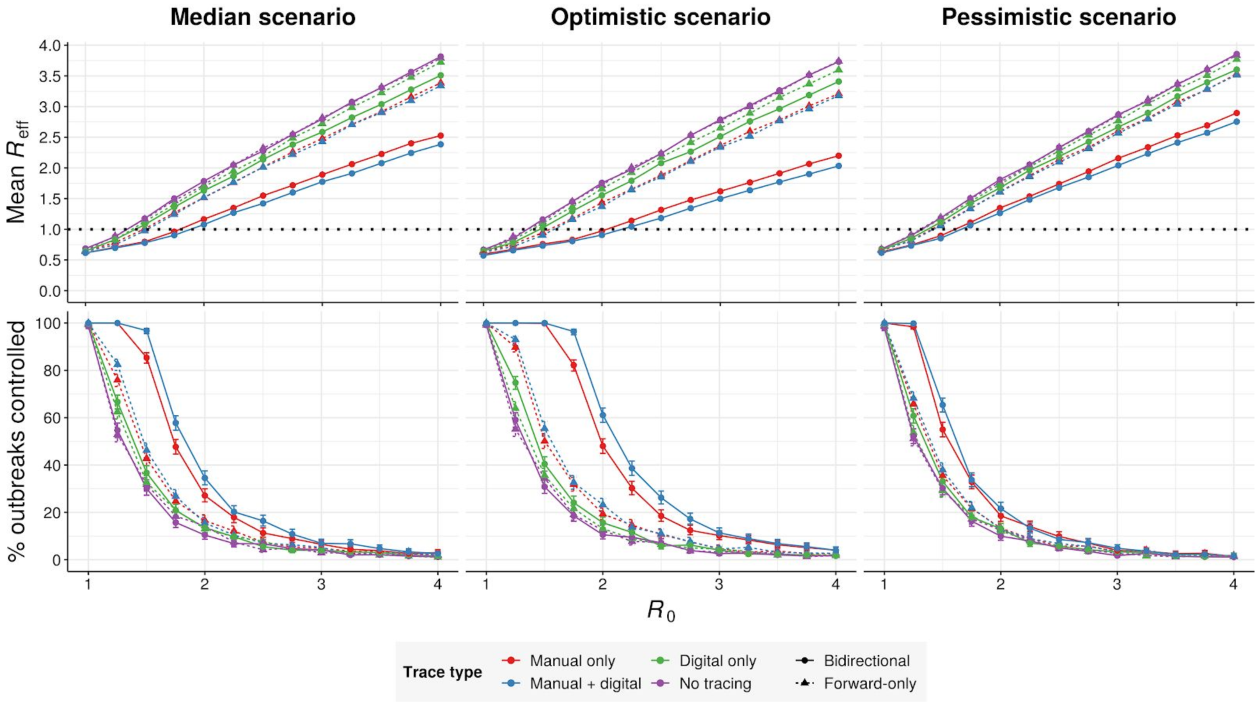

Supplementary Figure 9. Effect of R_0 and disease parameters on performance (low digital uptake). As Fig. 4, but assuming that 53% of cases have chirping smartphones. (top row) Mean R_{eff} achieved and (bottom row) % of outbreaks controlled by different tracing strategies as a function of the basic reproduction number R_0, assuming (left) median, (middle) optimistic or (right) pessimistic disease parameters (Supplementary Table 1). Error bars in the bottom row represent 95% credible intervals across 1000 runs under a uniform beta prior.

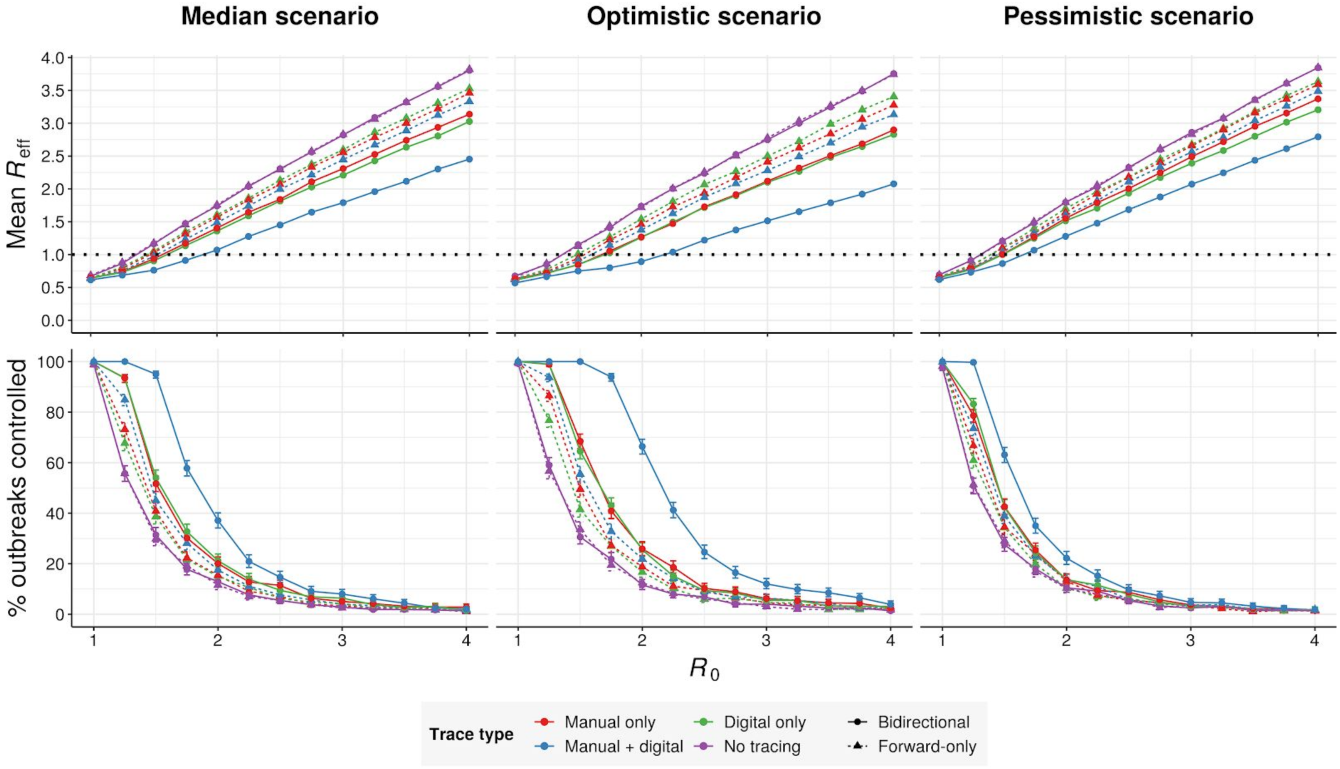

Supplementary Figure 10. Effect of R_0 and disease parameters on performance (2-day manual window). As Fig. 4, but assuming a 2-day manual tracing window. Note that the data-retention window for digital tracing is still 14 days. (top row) Mean R_{eff} achieved and (bottom row) % of outbreaks controlled by different tracing strategies as a function of the basic reproduction number R_0, assuming (left) median, (middle) optimistic or (right) pessimistic disease parameters (Supplementary Table 1). Error bars in the bottom row represent 95% credible intervals across 1000 runs under a uniform beta prior.

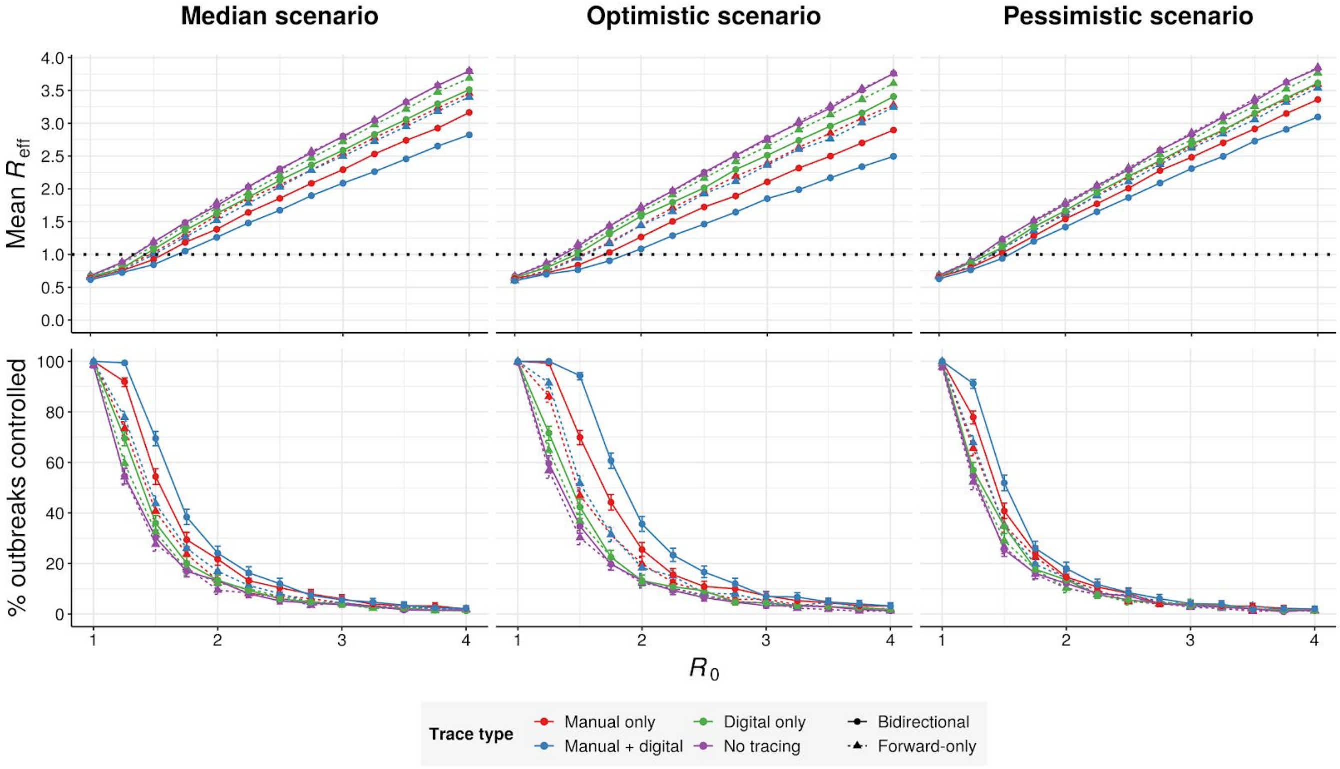

Supplementary Figure 11. Effect of R_0 and disease parameters on performance (2-day manual window, low digital uptake). As Fig. 4, but assuming a 2-day manual tracing window and that 53% of cases have chirping smartphones. Note that the data-retention window for digital tracing is still 14 days. (top row) Mean R_{eff} achieved and (bottom row) % of outbreaks controlled by different tracing strategies as a function of the basic reproduction number R_0, assuming (left) median, (middle) optimistic or (right) pessimistic disease parameters (Supplementary Table 1). Error bars in the bottom row represent 95% credible intervals across 1000 runs under a uniform beta prior.

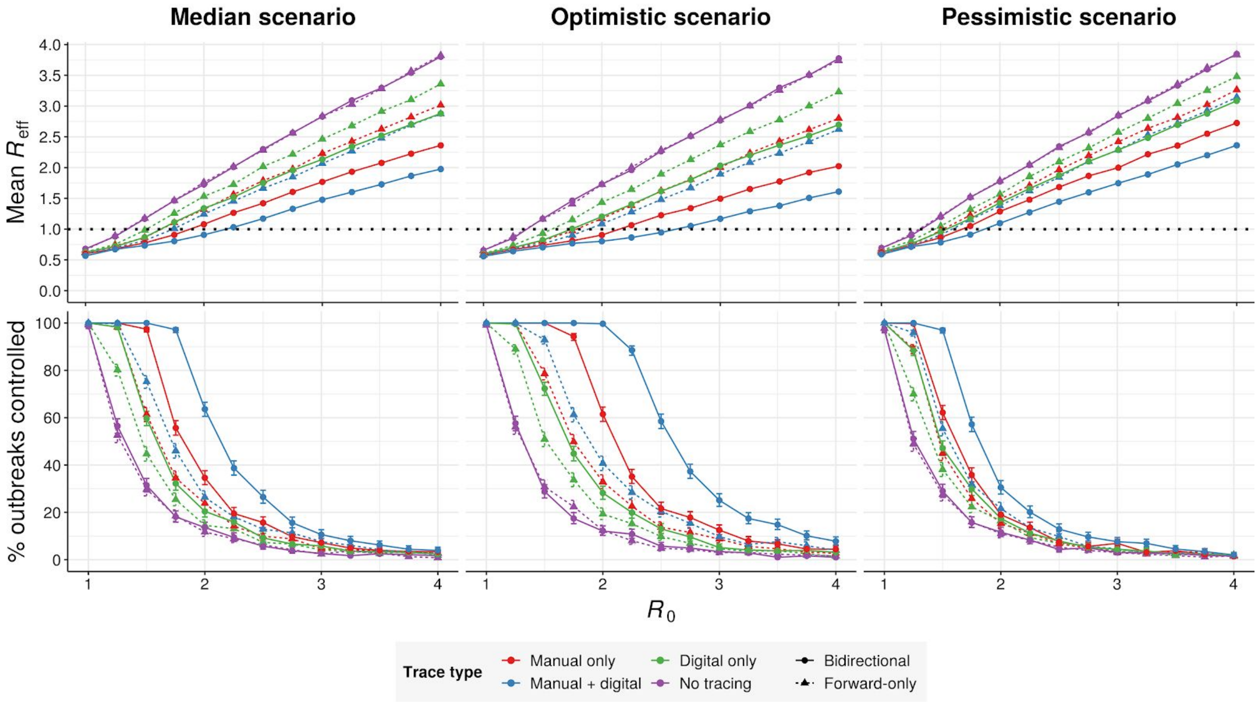

Supplementary Figure 12. Effect of R_0 and disease parameters on performance (no test required). As Fig. 4, but assuming that tracing could be initiated from identified symptomatic cases without a positive test result. (top row) Mean R_{eff} achieved and (bottom row) % of outbreaks controlled by different tracing strategies as a function of the basic reproduction number R_0, assuming (left) median, (middle) optimistic or (right) pessimistic disease parameters (Supplementary Table 1). Error bars in the bottom row represent 95% credible intervals across 1000 runs under a uniform beta prior.

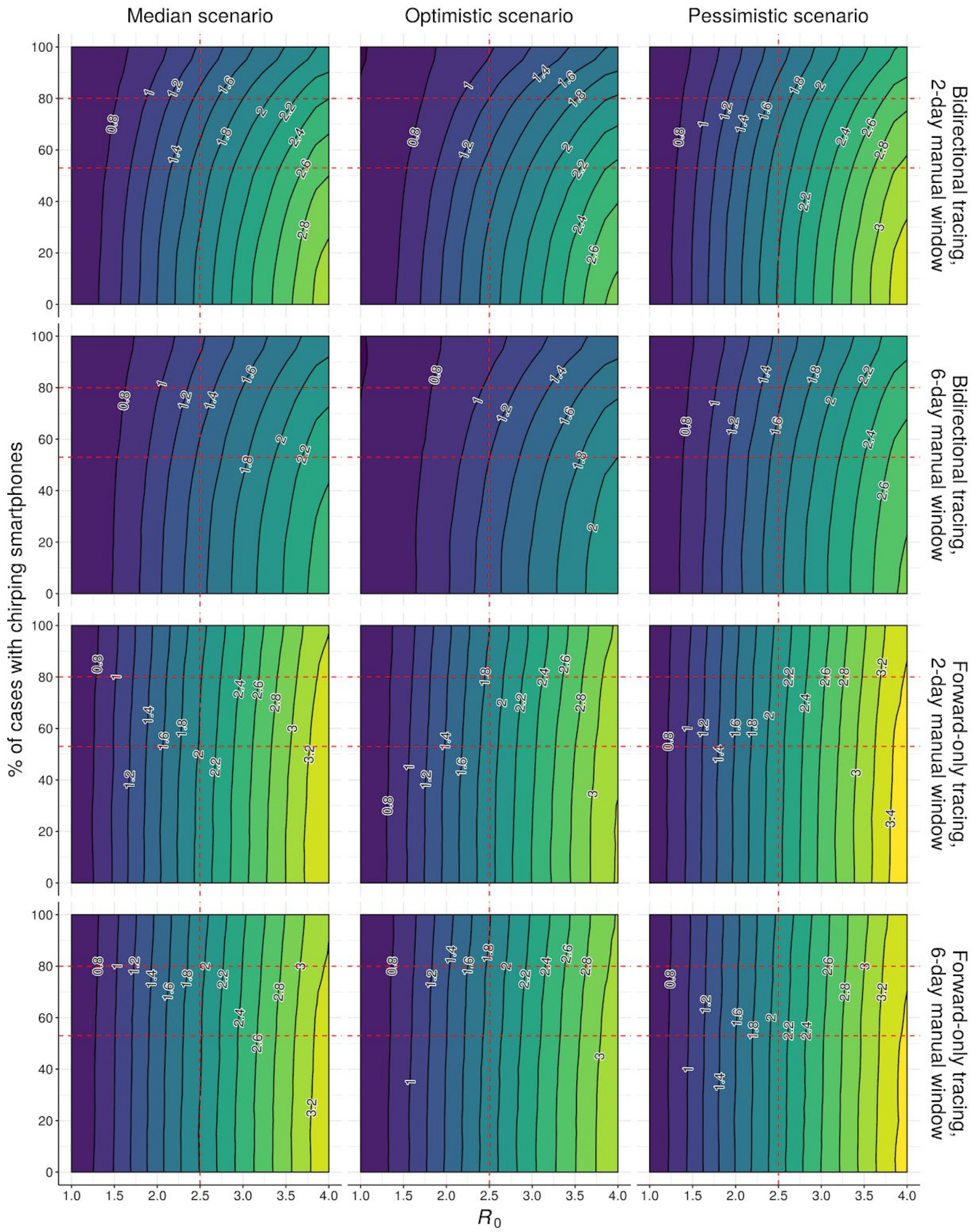

Supplementary Figure 13: Effect of R0, network fragmentation, and disease parameters on hybrid tracing performance ($\boldsymbol{R}_{\mathbf{eff}}$). Neighbour-averaged contour plots of the mean effective reproduction number achieved across 1000 runs per condition, under varying tracing strategies (panels), R_0 values (x-axis) and chirping coverage levels (y-axis), and assuming 90% probability of trace success and otherwise median disease parameters (Supplementary Table 1). Dashed red lines indicate values used in main-text figures.

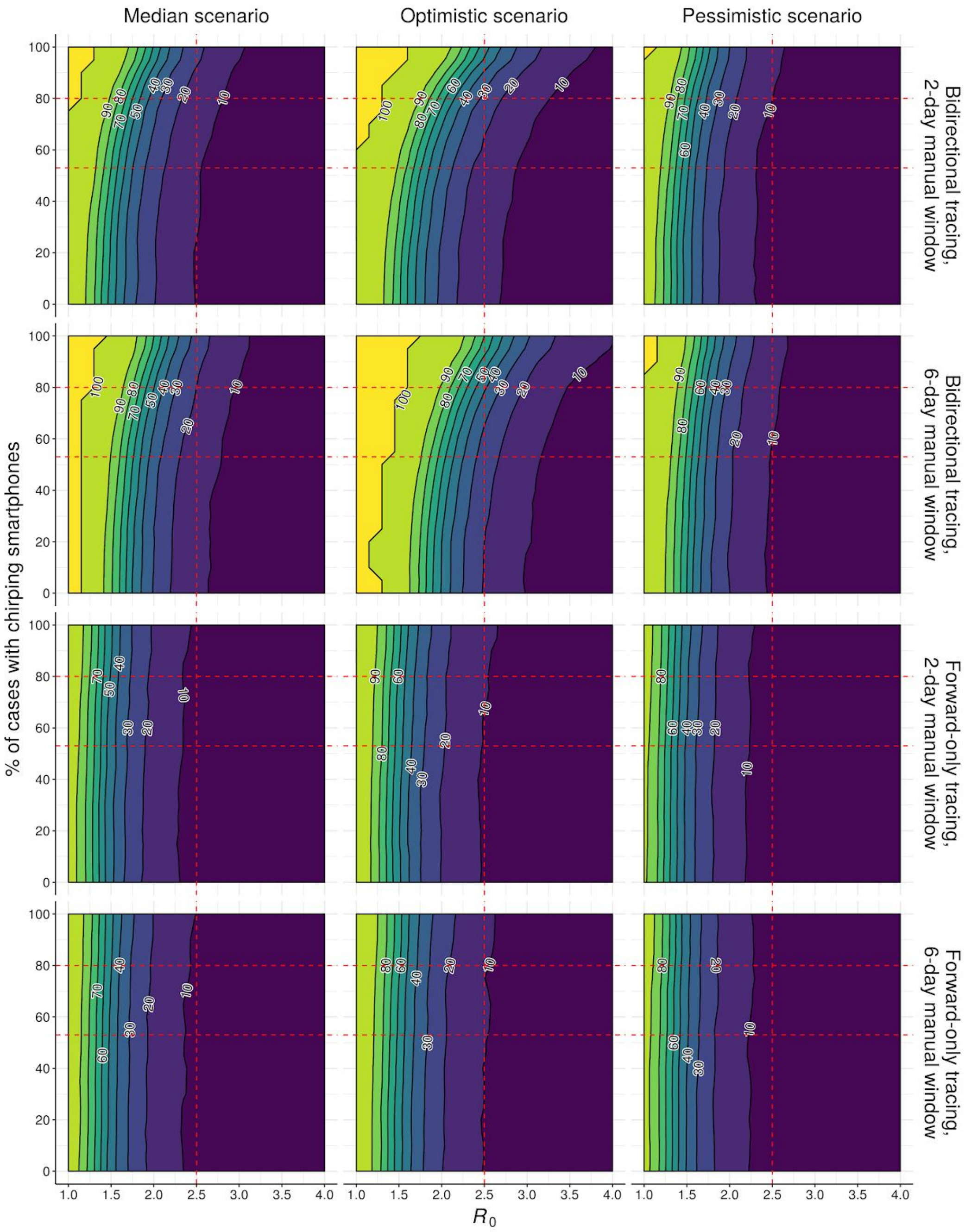

Supplementary Figure 14: Effect of R0, network fragmentation, and disease parameters on hybrid tracing performance (% outbreaks controlled). Neighbour-averaged contour plots of the % of outbreaks controlled across 1000 runs per condition, under varying tracing strategies (panels), R_0 values (x-axis) and chirping coverage levels (y-axis), and assuming 90% probability of trace success and otherwise median disease parameters (Supplementary Table 1). Dashed red lines indicate values used in main-text figures.

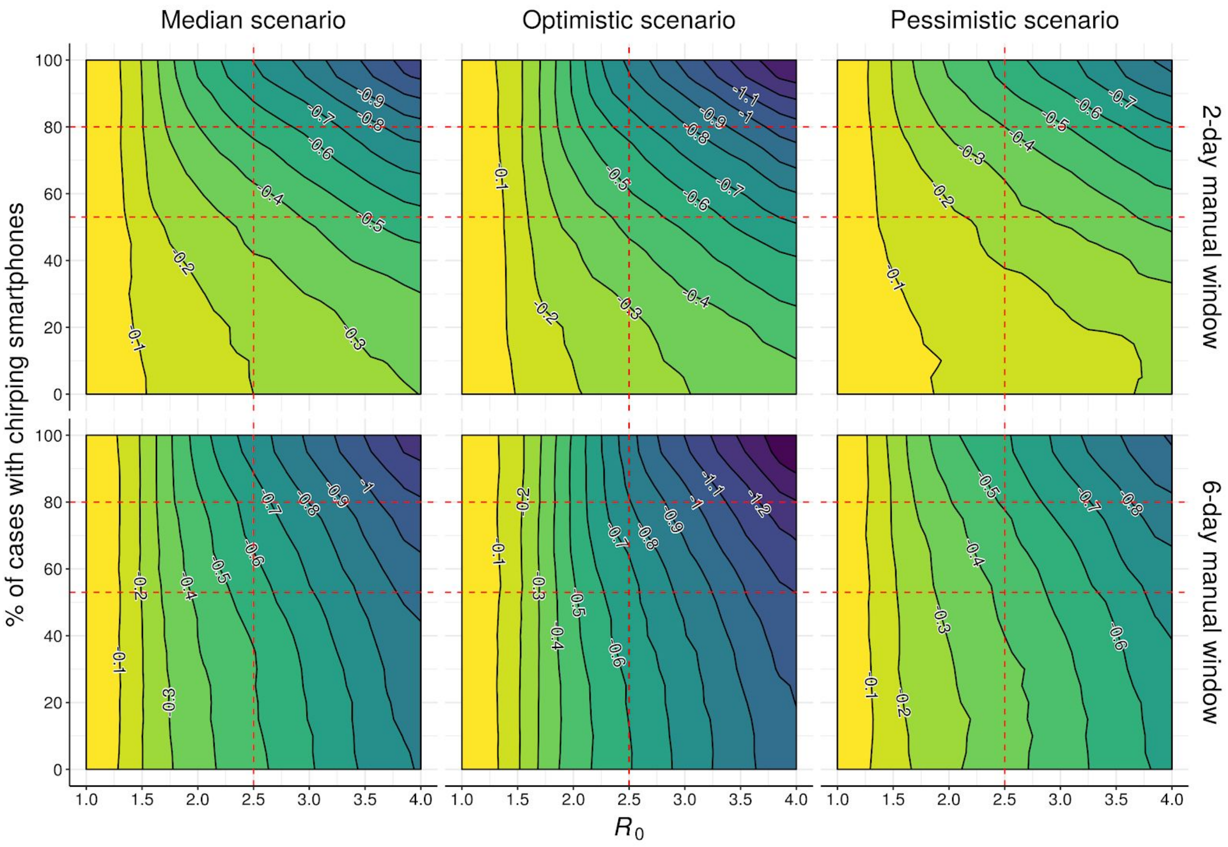

Supplementary Figure 15: Effect of R0, network fragmentation, and disease parameters on outperformance of bidirectional tracing (R_{eff}). Neighbour-averaged contour plots of the mean difference in effective reproduction number achieved by bidirectional relative to forward-only tracing, across 1000 runs per condition, under varying tracing strategies (panels), R_0 values (x-axis) and chirping coverage levels (y-axis), and assuming 90% probability of trace success and otherwise median disease parameters (Supplementary Table 1). Dashed red lines indicate values used in main-text figures.

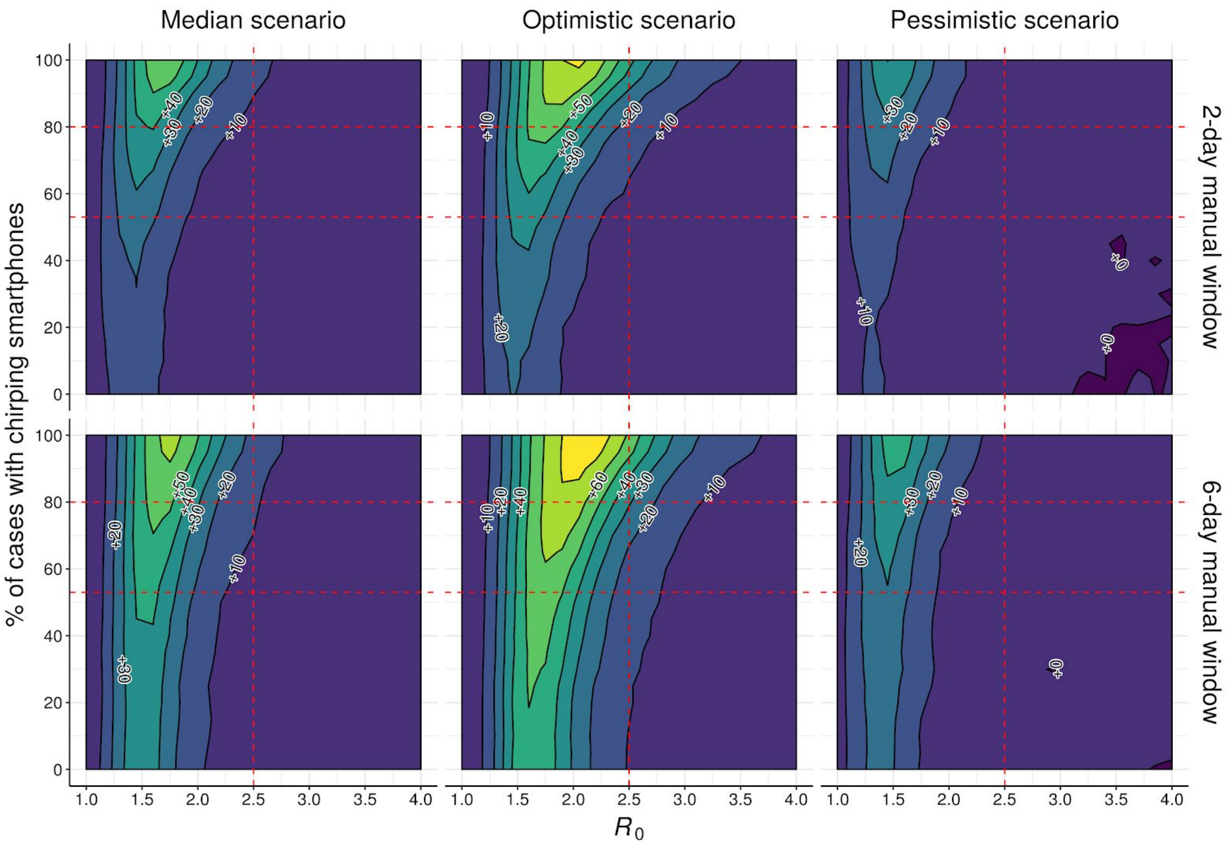

Supplementary Figure 16: Effect of R0, network fragmentation, and disease parameters on outperformance of bidirectional tracing (% outbreaks controlled). Neighbour-averaged contour plots of the mean difference in % of outbreaks controlled by bidirectional relative to forward-only tracing, across 1000 runs per condition, under varying tracing strategies (panels), R_0 values (x-axis) and chirping coverage levels (y-axis), and assuming 90% probability of trace success and otherwise median disease parameters (Supplementary Table 1). Dashed red lines indicate values used in main-text figures.

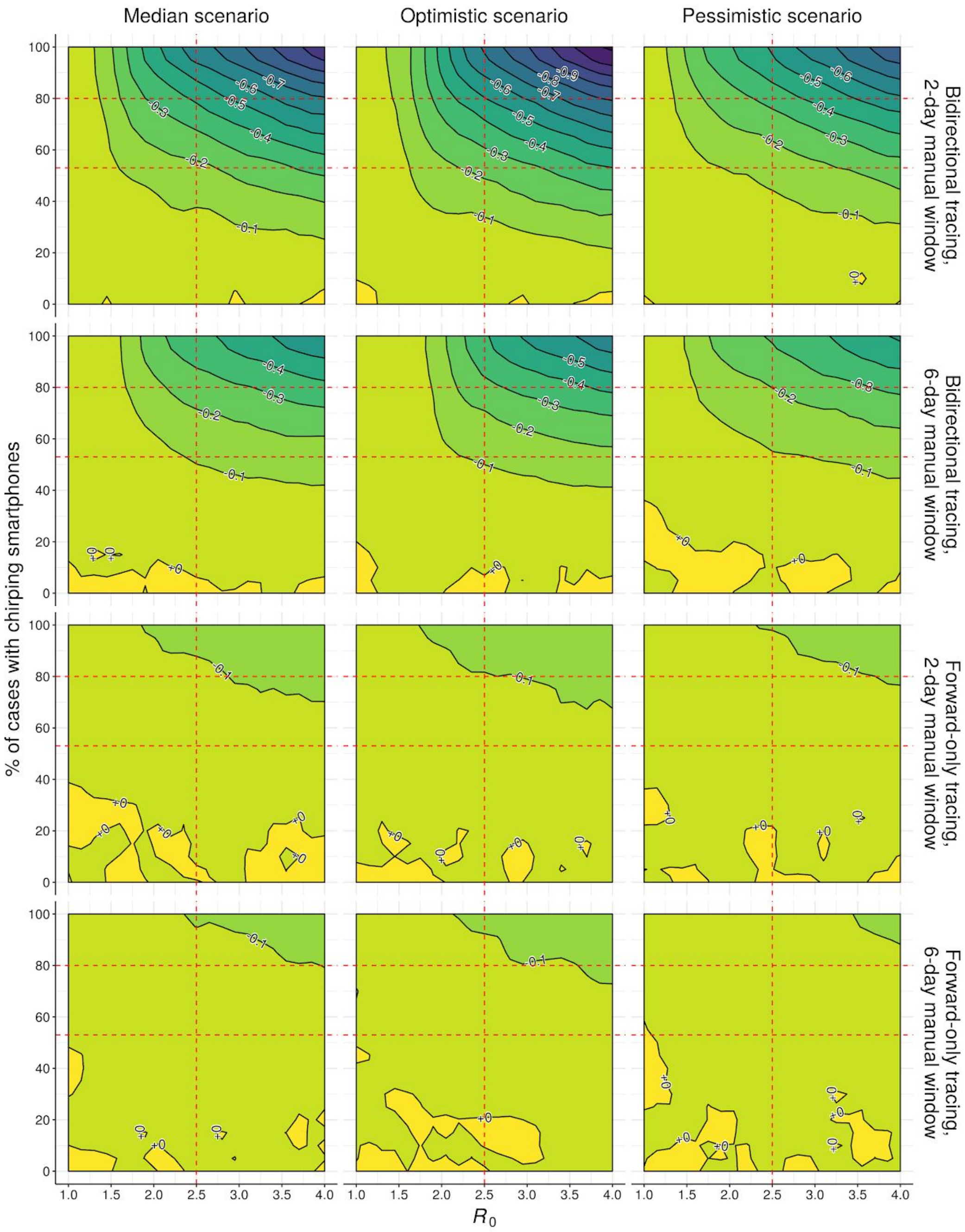

Supplementary Figure 17: Effect of R0, network fragmentation, and disease parameters on outperformance of hybrid tracing (R_{eff}). Neighbour-averaged contour plots of the mean difference in effective reproduction number achieved by hybrid relative to manual tracing, across 1000 runs per condition, under varying tracing strategies (panels), R_0 values (x-axis) and chirping coverage levels (y-axis), and assuming 90% probability of trace success and otherwise median disease parameters (Supplementary Table 1). Dashed red lines indicate values used in main-text figures.

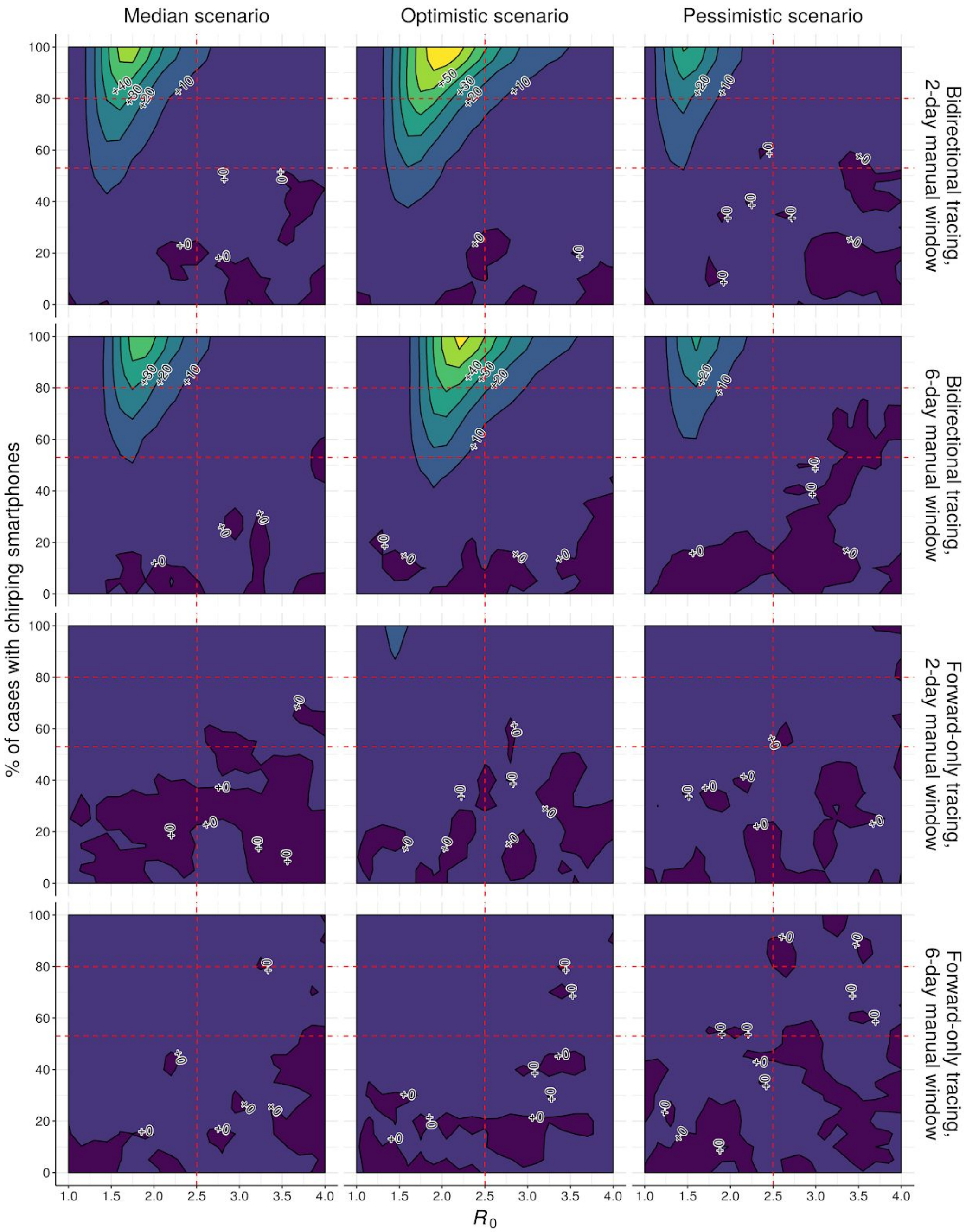

Supplementary Figure 18: Effect of R0, network fragmentation, and disease parameters on outperformance of hybrid tracing (% outbreaks controlled). Neighbour-averaged contour plots of the mean difference in % of outbreaks controlled by hybrid relative to manual tracing, across 1000 runs per condition, under varying tracing strategies (panels), R_0 values (x-axis) and chirping coverage levels (y-axis), and assuming 90% probability of trace success and otherwise median disease parameters (Supplementary Table 1). Dashed red lines indicate values used in main-text figures.

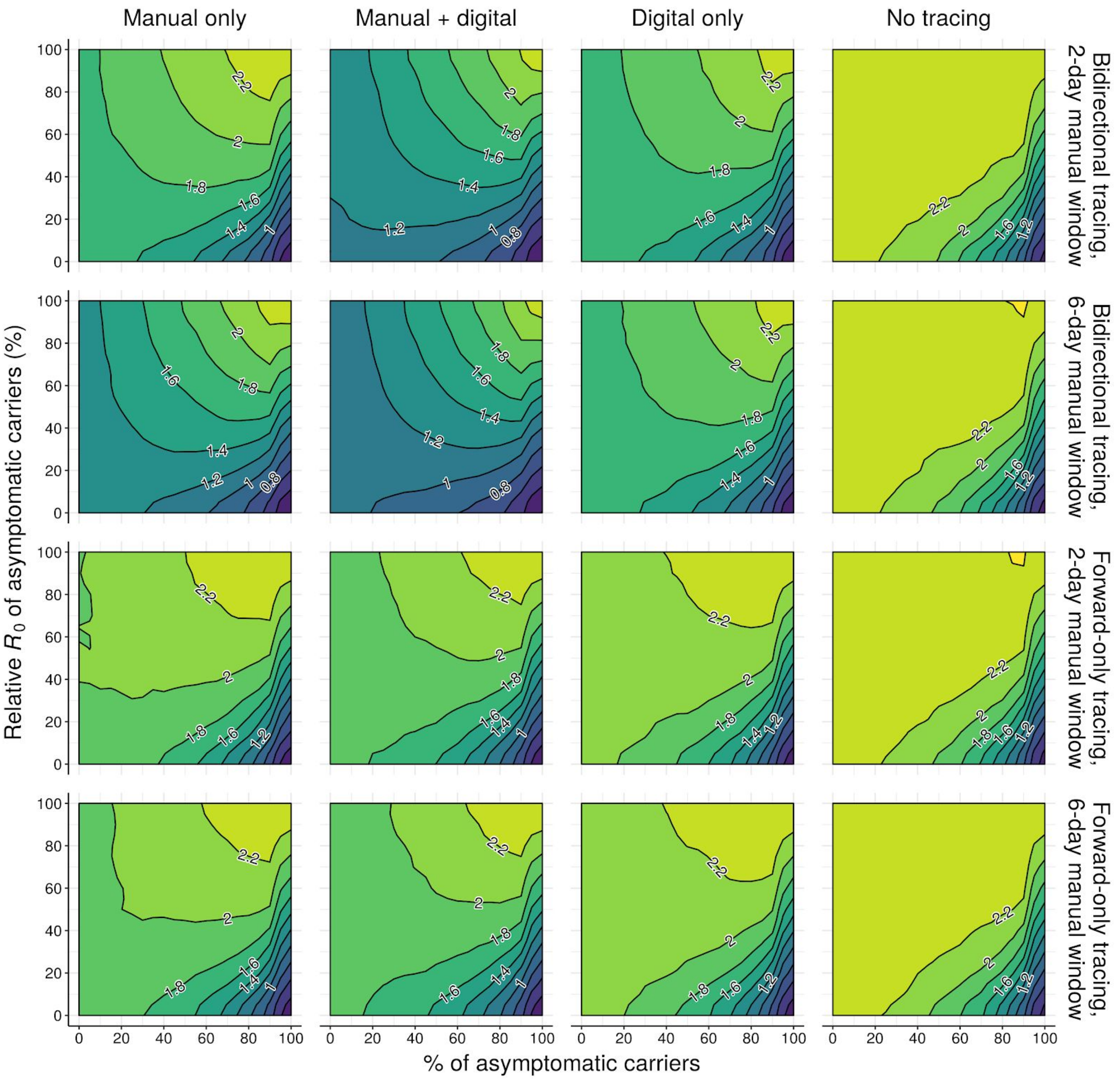

Supplementary Figure 19: Effect of the rate and infectiousness of asymptomatic carriers on tracing performance (R_{eff}, high digital uptake). Neighbour-averaged contour plots of the mean effective reproduction number achieved across 500 runs per condition, under varying tracing strategies (panels), frequencies of asymptomatic carriers (x-axis) and relative infectiousness of asymptomatic carriers (y-axis), and assuming 90% probability of trace success, high (80%) digital uptake, and otherwise median disease parameters (Supplementary Table 1).

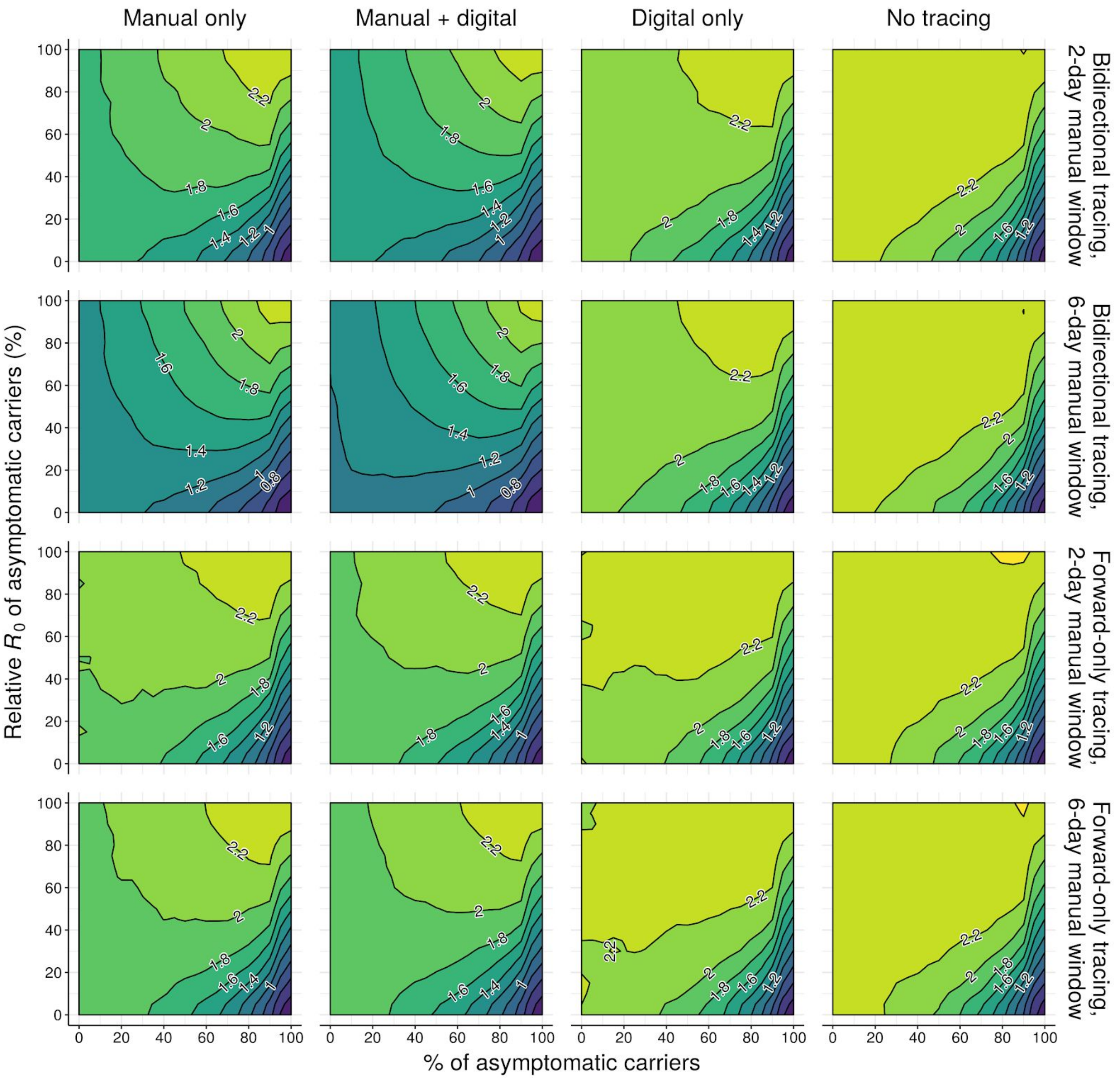

Supplementary Figure 20: Effect of the rate and infectiousness of asymptomatic carriers on tracing performance (R_{eff}, low digital uptake). Neighbour-averaged contour plots of the mean effective reproduction number achieved across 500 runs per condition, under varying tracing strategies (panels), frequencies of asymptomatic carriers (x-axis) and relative infectiousness of asymptomatic carriers (y-axis), and assuming 90% probability of trace success, low (53%) digital uptake, and otherwise median disease parameters (Supplementary Table 1).

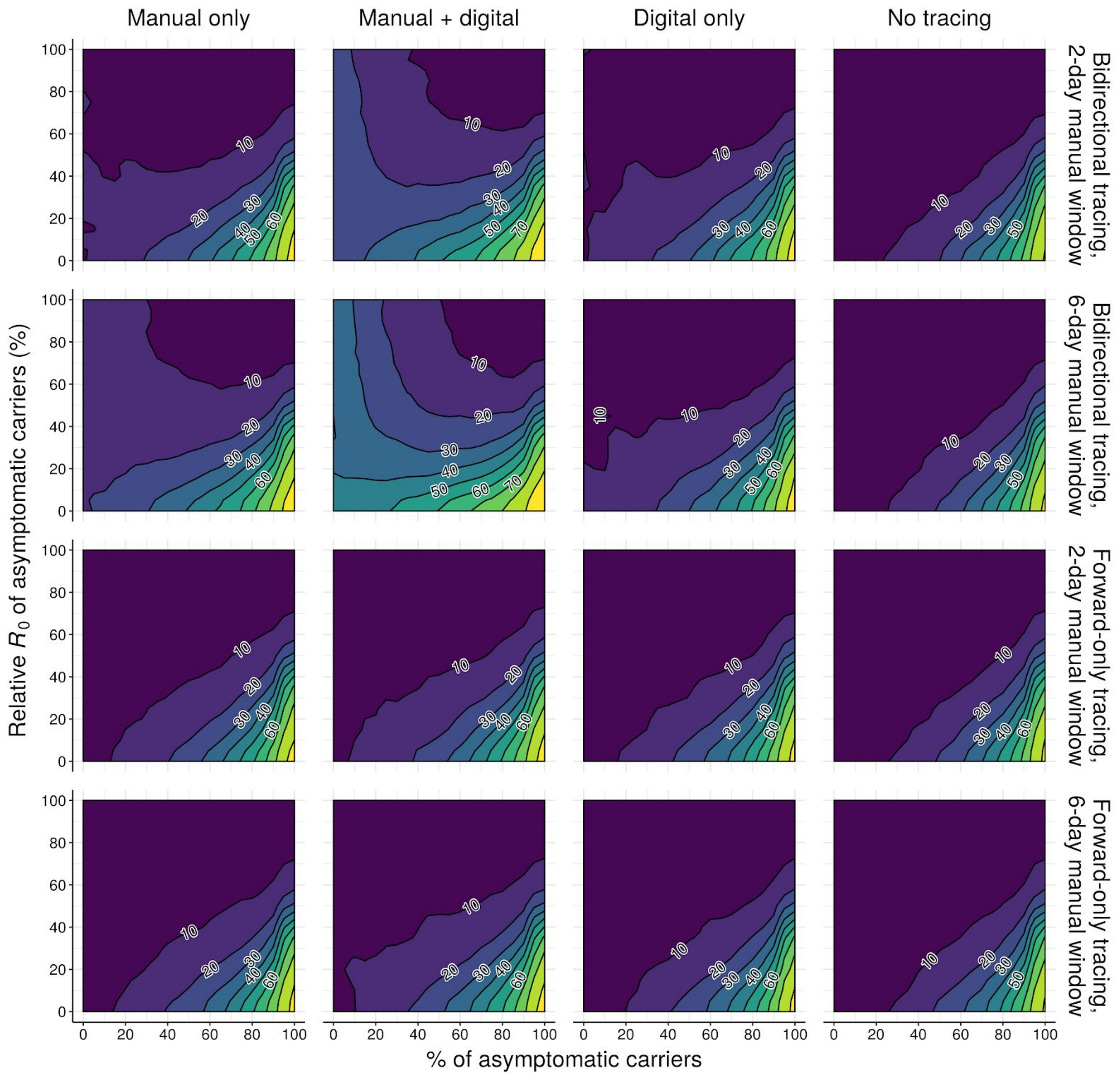

Supplementary Figure 21: Effect of the rate and infectiousness of asymptomatic carriers on tracing performance (% outbreaks controlled, high digital uptake). Neighbour-averaged contour plots of the % of outbreaks controlled across 500 runs per condition, under varying tracing strategies (panels), frequencies of asymptomatic carriers (x-axis) and relative infectiousness of asymptomatic carriers (y-axis), and assuming 90% probability of trace success, high (80%) digital uptake, and otherwise median disease parameters (Supplementary Table 1).

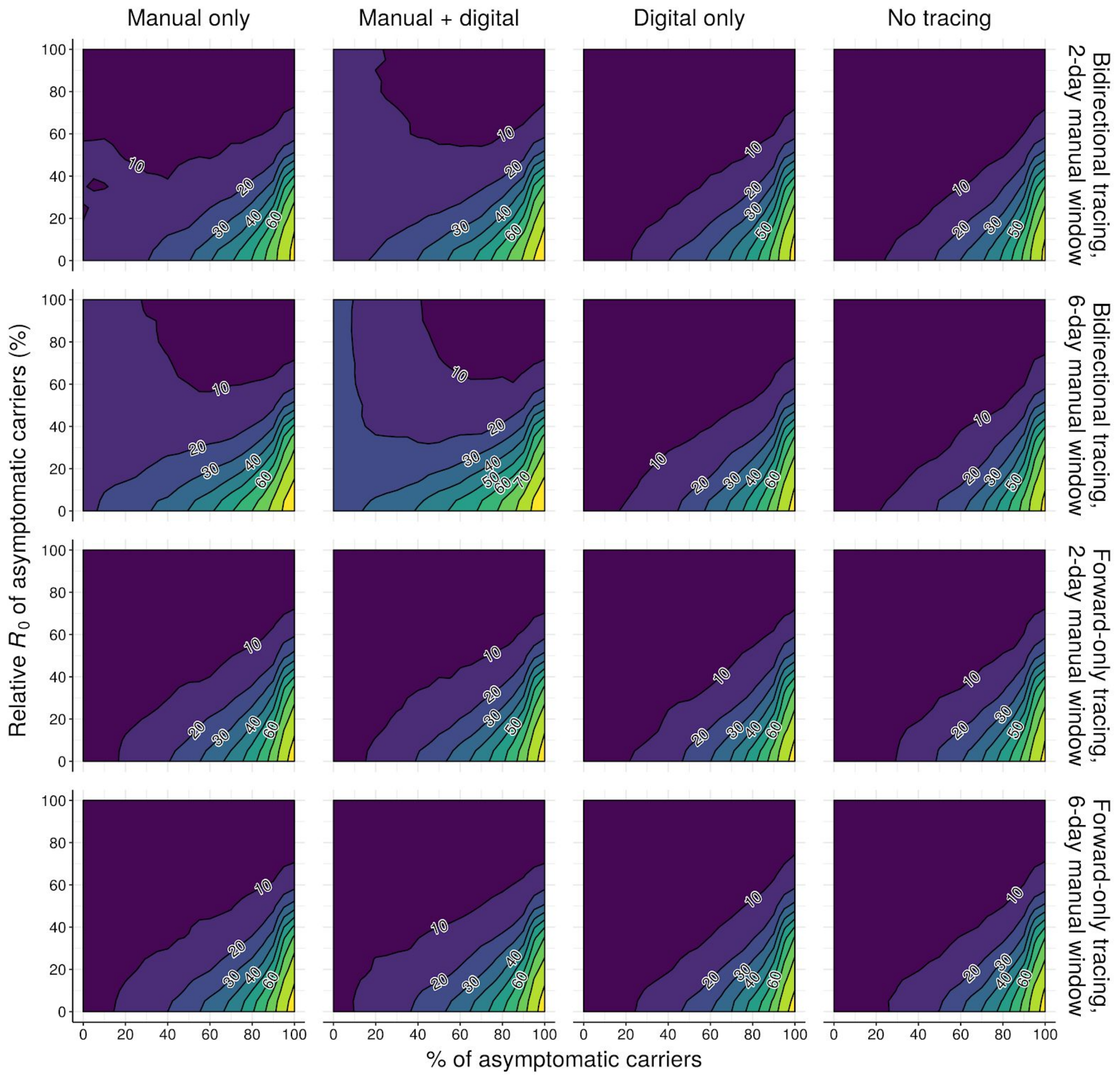

Supplementary Figure 22: Effect of the rate and infectiousness of asymptomatic carriers on tracing performance (% outbreaks controlled, low digital uptake). Neighbour-averaged contour plots of the % of outbreaks controlled across 500 runs per condition, under varying tracing strategies (panels), frequencies of asymptomatic carriers (x-axis) and relative infectiousness of asymptomatic carriers (y-axis), and assuming 90% probability of trace success, low (53%) digital uptake, and otherwise median disease parameters (Supplementary Table 1).

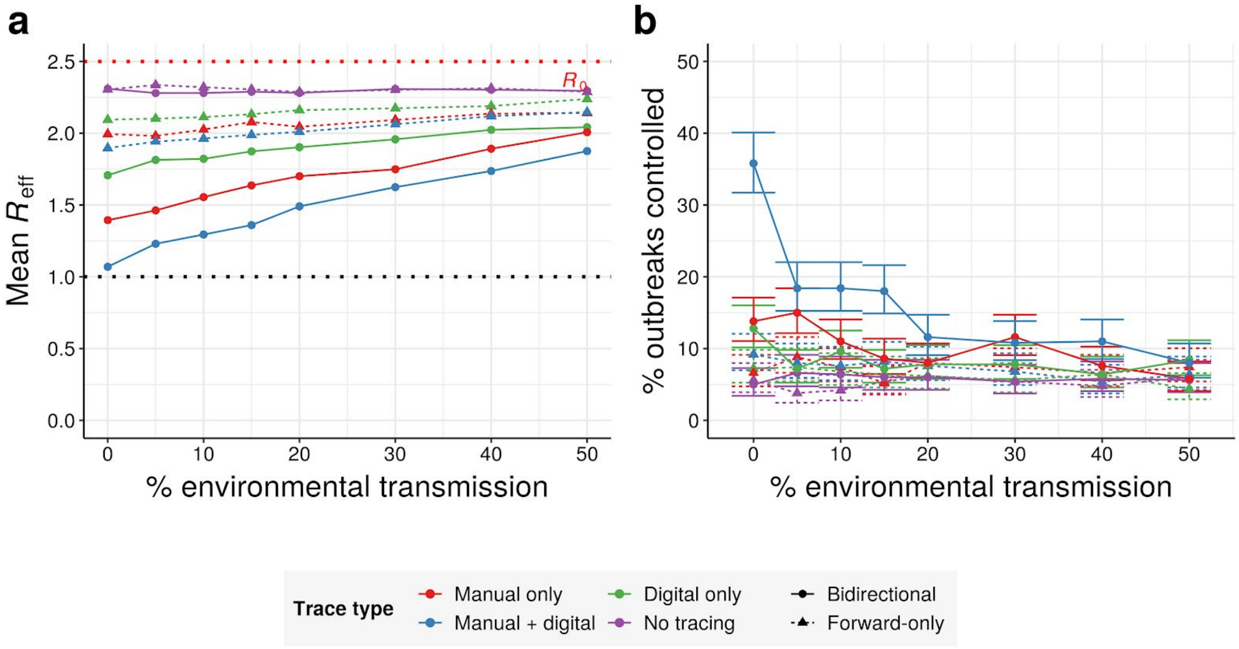

Supplementary Figure 23: Effect of environmental transmission on epidemic control. (a) Mean effective reproduction number achieved and (b) % of outbreaks controlled under different rates of environmental transmission, assuming 90% probability of trace success, a 6-day manual tracing window, and otherwise median disease parameters (Supplementary Table 1). Error bars in (b) represent 95% credible intervals across 500 runs under a uniform beta prior; points in (a) represent average values over the same. Note that "probability of trace success" excludes traces blocked by environmental transmission or network fragmentation.

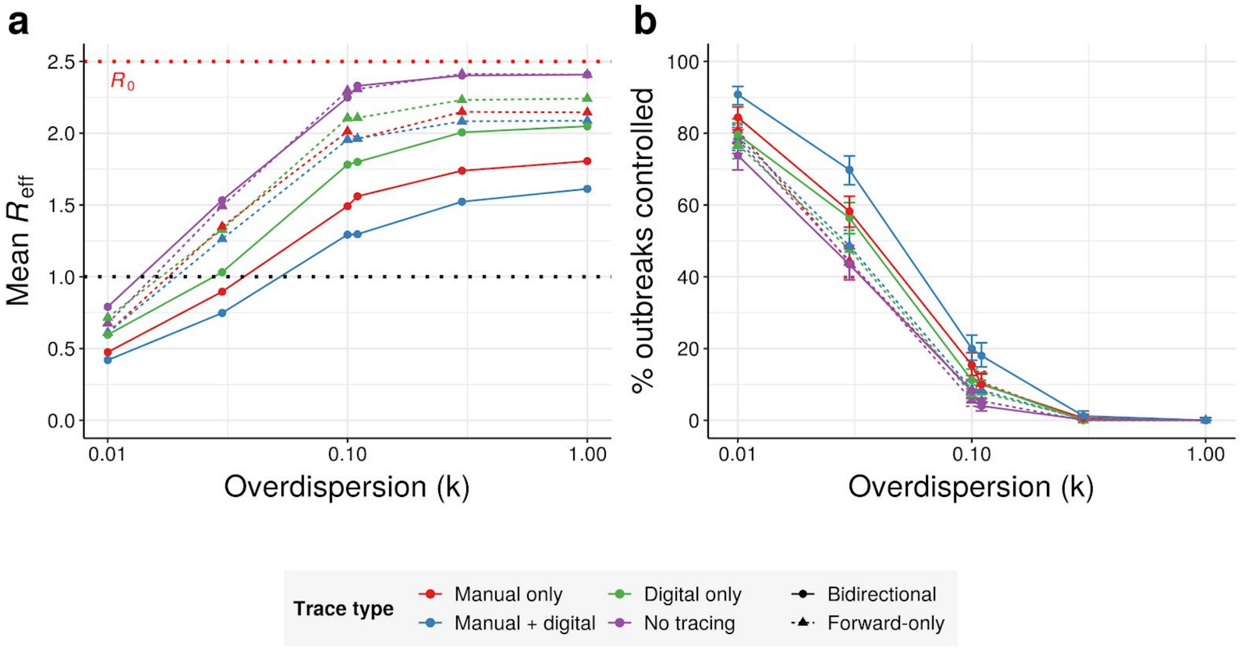

Supplementary Figure 24: Effect of overdispersion on epidemic control. (a) Mean effective reproduction number achieved and (b) % of outbreaks controlled under different values of the overdispersion parameter *k* (where lower values of k denote higher variance in numbers of secondary infections of cases), assuming 90% probability of trace success, a 6-day manual tracing window, and otherwise median disease parameters (Supplementary Table 1). Error bars in (b) represent 95% credible intervals across 500 runs under a uniform beta prior; points in (a) represent average values over the same. Note that "probability of trace success" excludes traces blocked by environmental transmission or network fragmentation.

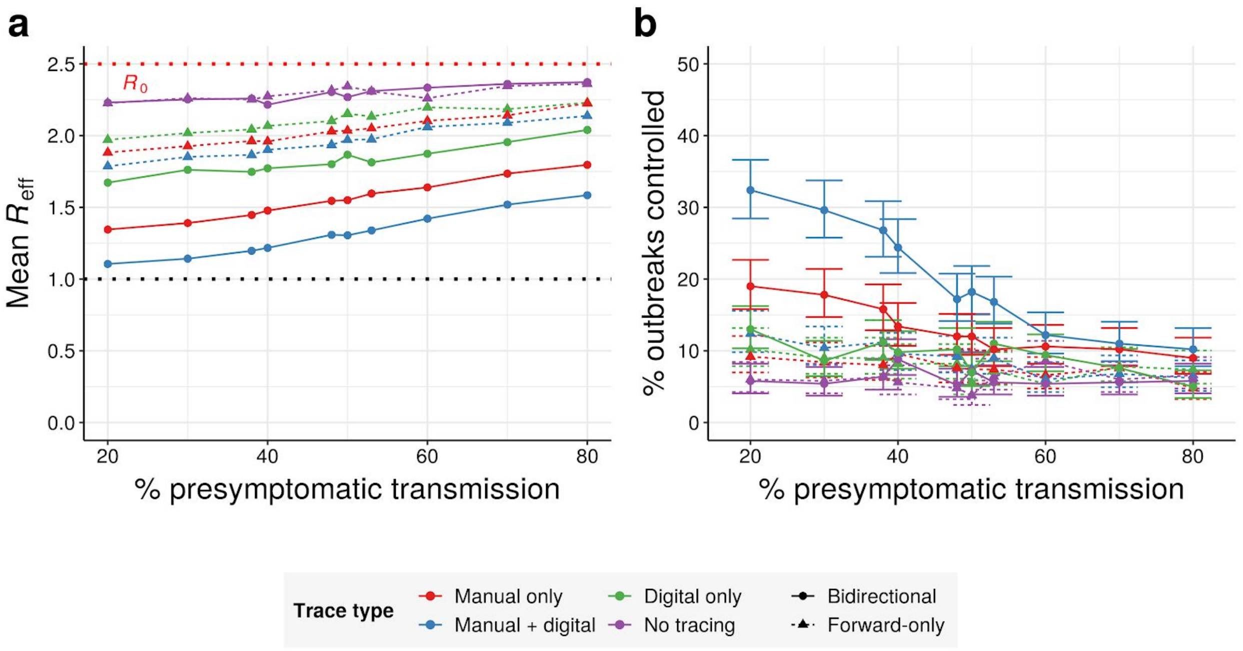

Supplementary Figure 25: Effect of presymptomatic transmission on epidemic control. (a) Mean effective reproduction number achieved and (b) % of outbreaks controlled under different rates of presymptomatic transmission, assuming 90% probability of trace success, a 6-day manual tracing window, and otherwise median disease parameters (Supplementary Table 1). Error bars in (b) represent 95% credible intervals across 500 runs under a uniform beta prior; points in (a) represent average values over the same.. Note that “probability of trace success” excludes traces blocked by environmental transmission or network fragmentation. See Supplementary Figure 33 for more on the relationship between the α-parameter of the generation-time distribution and the rate of presymptomatic transmission.

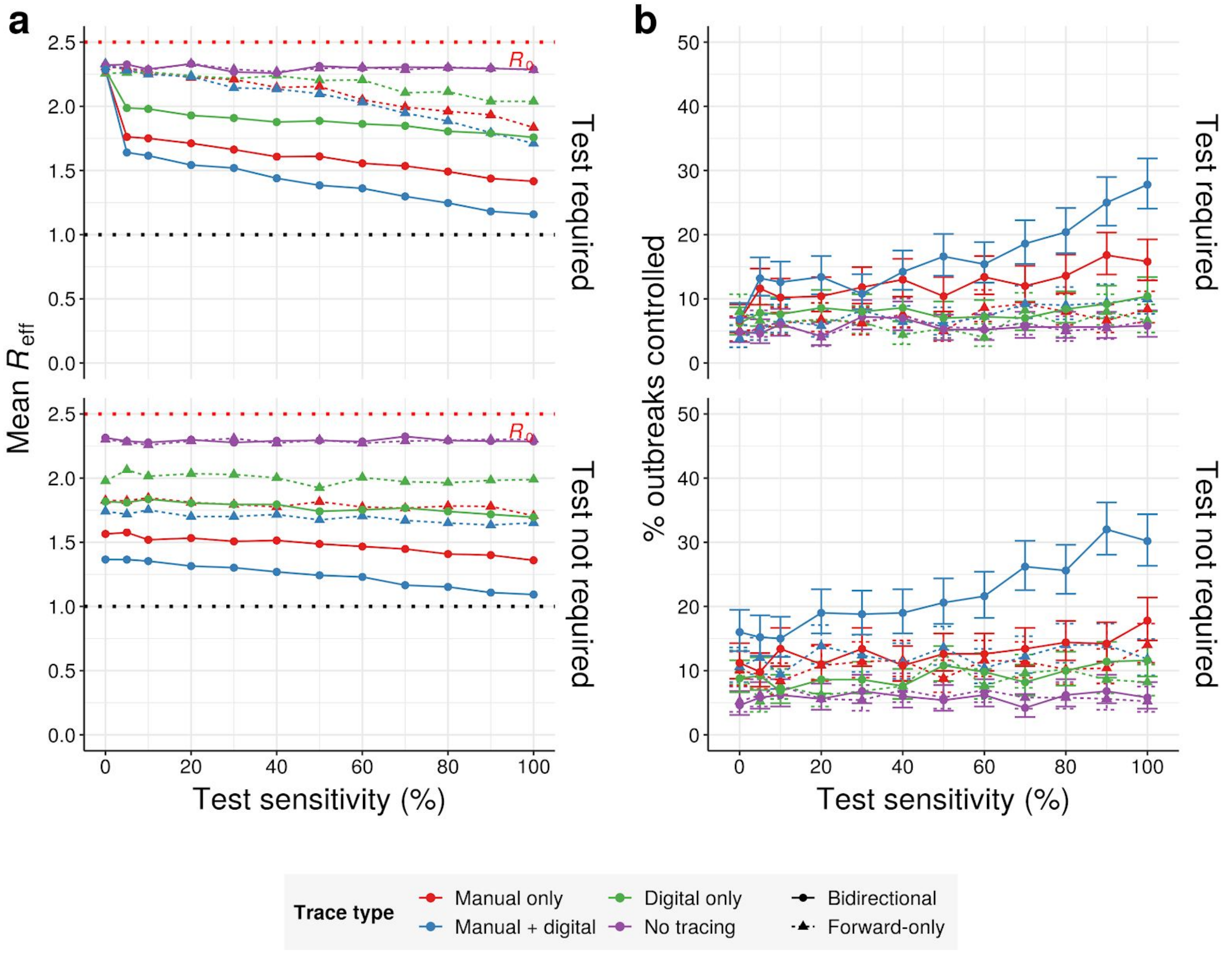

Supplementary Figure 26: Effect of test sensitivity on epidemic control. (a) Mean effective reproduction number achieved and (b) % of outbreaks controlled under different levels of test sensitivity, assuming 90% probability of trace success, a 6-day manual tracing window, and otherwise median disease parameters (Supplementary Table 1). Error bars in (b) represent 95% credible intervals across 500 runs under a uniform beta prior; points in (a) represent average values over the same. "Test required" indicates that a positive test result was required before initiating contact tracing from a symptomatic case, while "test not required" indicates that tracing could be initiated based on symptoms alone. Note that "probability of trace success" excludes traces blocked by environmental transmission or network fragmentation.

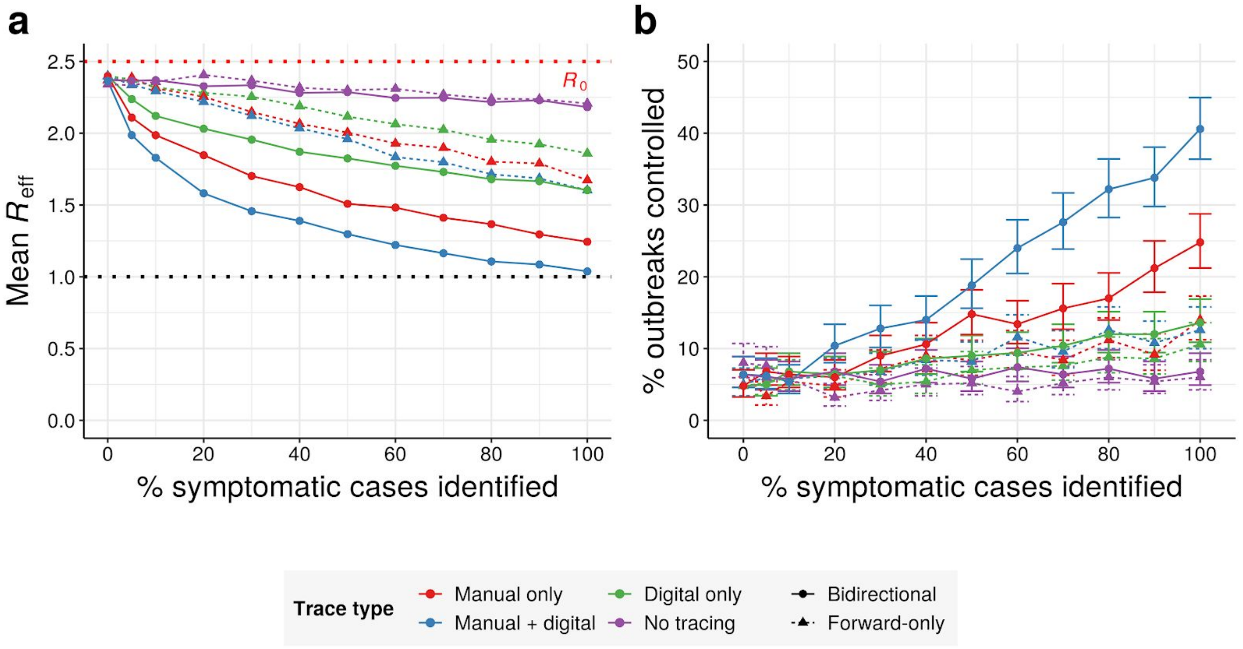

Supplementary Figure 27: Effect of case ascertainment on epidemic control. (a) Mean effective reproduction number achieved and (b) % of outbreaks controlled under different levels of ascertainment of symptomatic cases, assuming 90% probability of trace success, a 6-day manual tracing window, and otherwise median disease parameters (Supplementary Table 1). Error bars in (b) represent 95% credible intervals across 500 runs under a uniform beta prior; points in (a) represent average values over the same. Note that "probability of trace success" excludes traces blocked by environmental transmission or network fragmentation. Note also that, since 45% of cases are asymptomatic in our median scenario (and thus never identified from symptoms), overall ascertainment when x% of symptomatic cases are identified is roughly $0.55x$%.

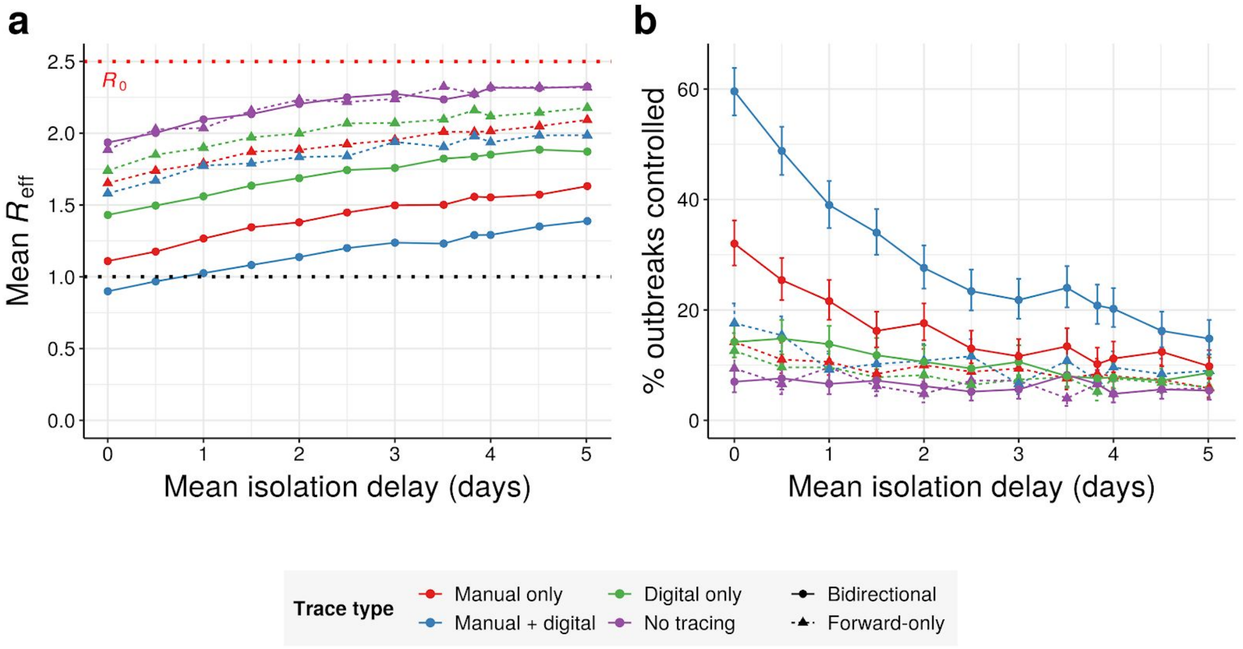

Supplementary Figure 28: Effect of case isolation delay time on epidemic control. (a) Mean effective reproduction number achieved and (b) % of outbreaks controlled as a function of the mean time from symptom onset to isolation for symptomatic cases (based on symptoms alone), assuming 90% probability of trace success, a 6-day manual tracing window, and otherwise median disease parameters (Supplementary Table 1). Error bars in (b) represent 95% credible intervals across 500 runs under a uniform beta prior; points in (a) represent average values over the same. Note that "probability of trace success" excludes traces blocked by environmental transmission or network fragmentation.

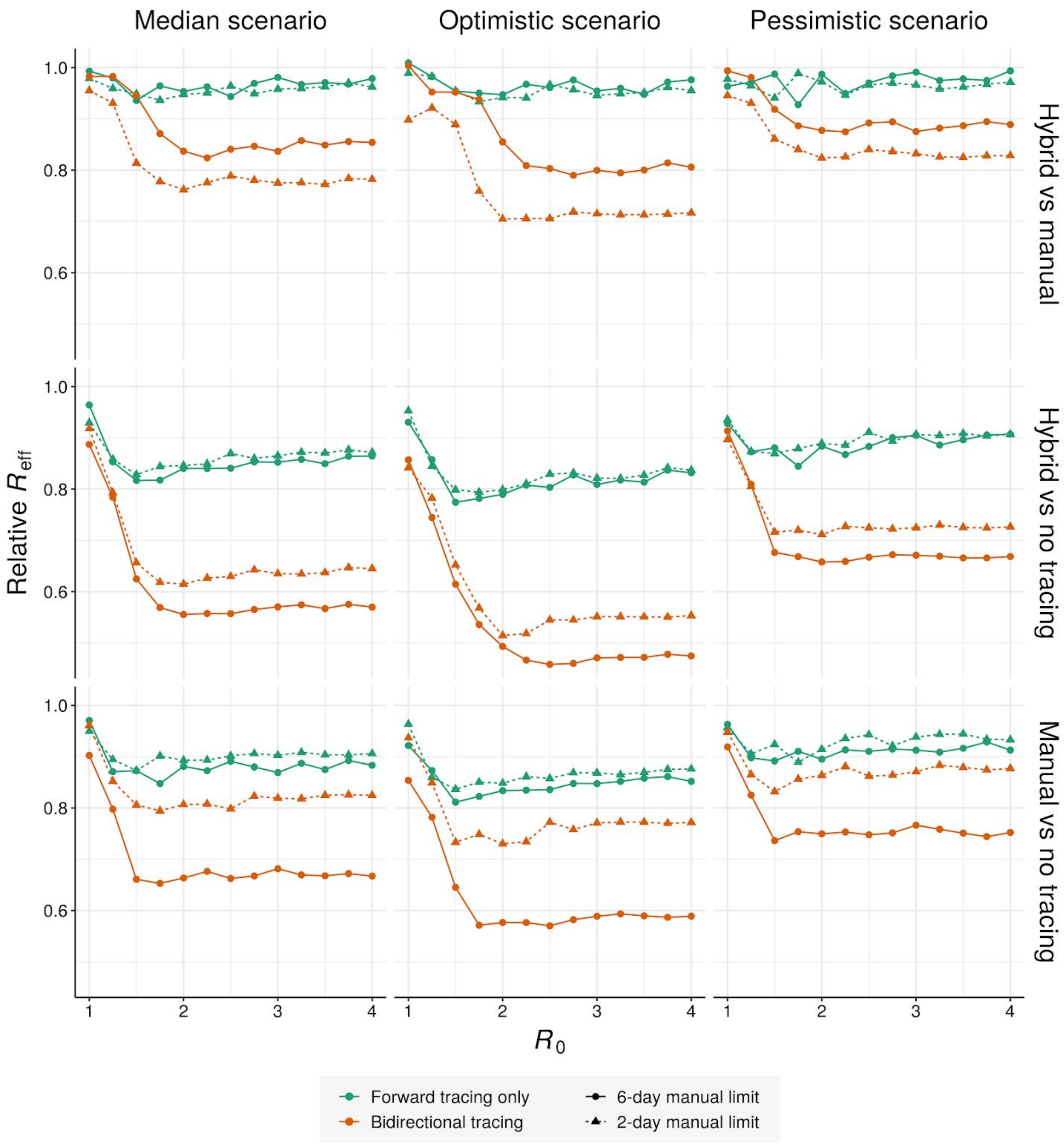

Supplementary Figure 29: Relative performance of hybrid, manual and no tracing as a function of R_0. Ratio between the effective reproduction number (over 1000 runs per condition) achieved by (top row) hybrid vs manual tracing, (middle row) hybrid vs no tracing, and (bottom row) manual vs no tracing, under (left) median, (middle) optimistic and (right) pessimistic disease parameters (Supplementary Table 1), assuming 90% probability of trace success and high uptake of the digital system.

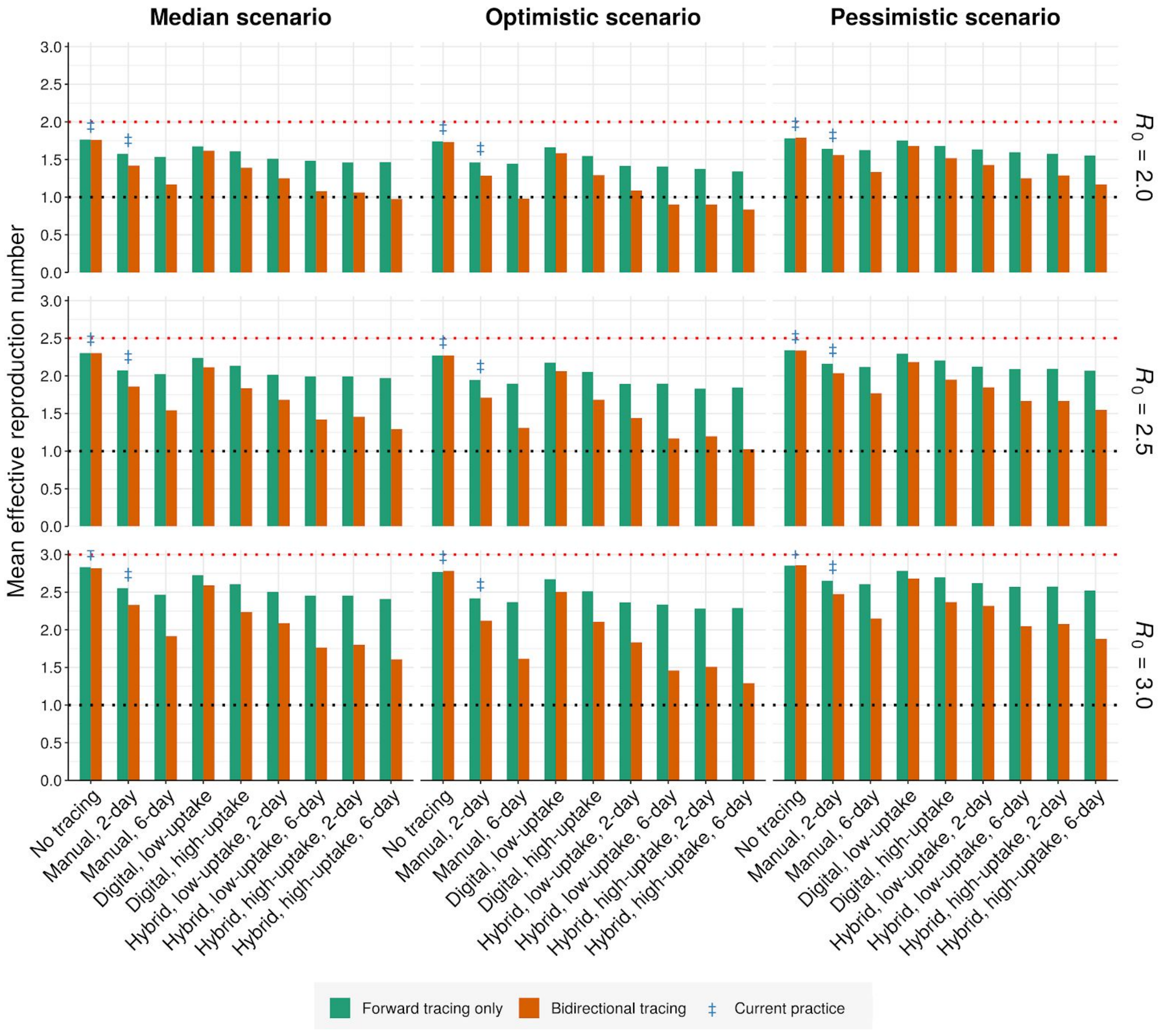

Supplementary Figure 30. Performance of different tracing strategies for controlling COVID-19 (varying R_0). As Fig. 5, but including results for R_0 = 2.0 and R_0 = 3.0. Mean effective reproduction number obtained under (left) median, (middle) optimistic, and (right) pessimistic scenarios (Supplementary Table 1), assuming a 90% baseline probability of trace success across 1000 runs. Blue double dagger symbols indicate conditions roughly corresponding to current practice in most regions. Low and high uptake correspond to 53% and 80% of cases, respectively, having chirp-enabled smartphones. Without tracing, forward and bidirectional are equivalent.

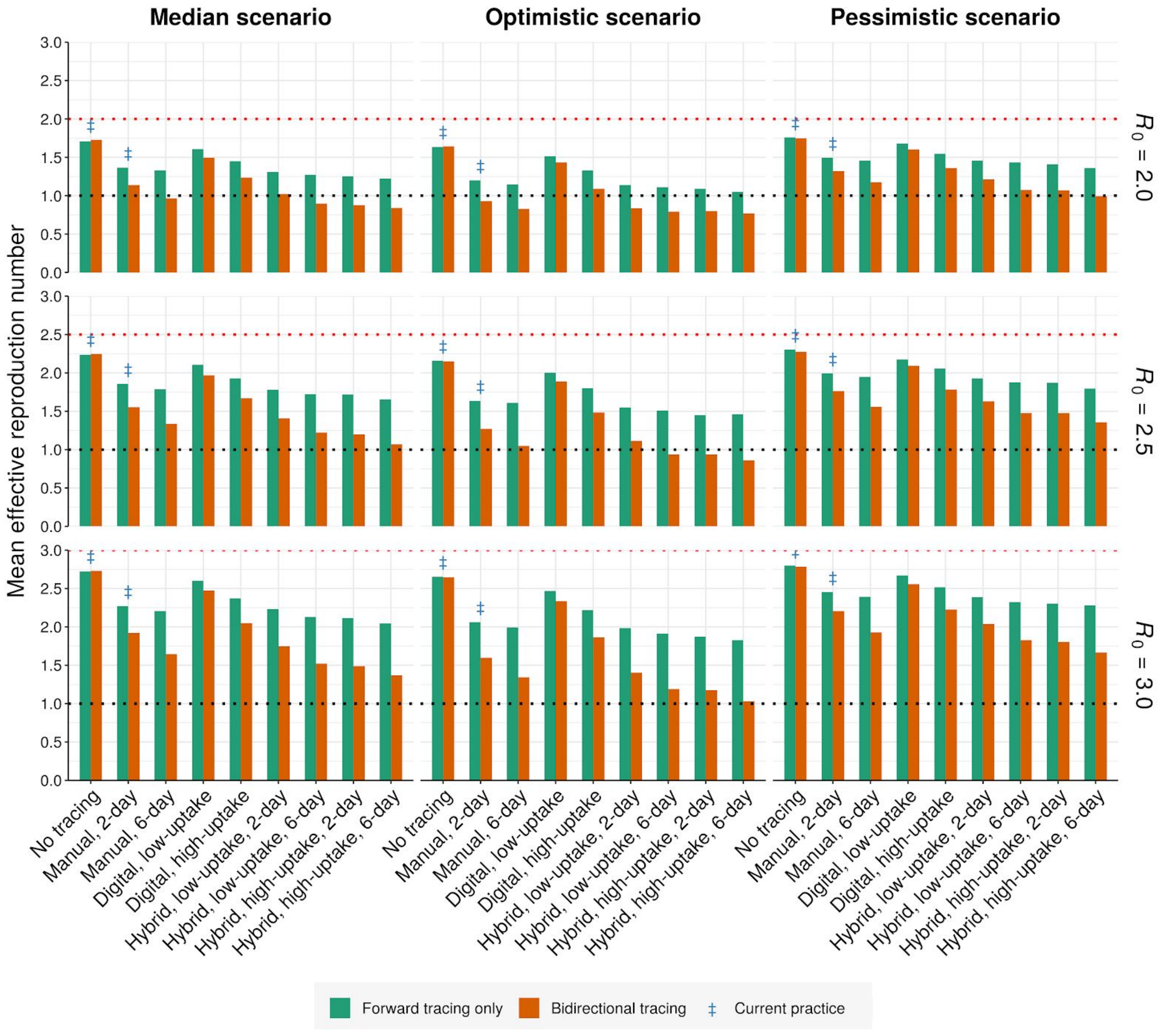

Supplementary Figure 31. Performance of different tracing strategies for controlling COVID-19 (high ascertainment). As Supplementary Fig. 30, but assuming that 90% of symptomatic cases can be detected based on symptoms alone. Mean effective reproduction number obtained under (left) median, (middle) optimistic, and (right) pessimistic scenarios (Supplementary Table 1), assuming a 90% baseline probability of trace success across 1000 runs. Blue double dagger symbols indicate conditions roughly corresponding to current practice in most regions. Low and high uptake correspond to 53% and 80% of cases, respectively, having chirp-enabled smartphones. Without tracing, forward and bidirectional are equivalent.

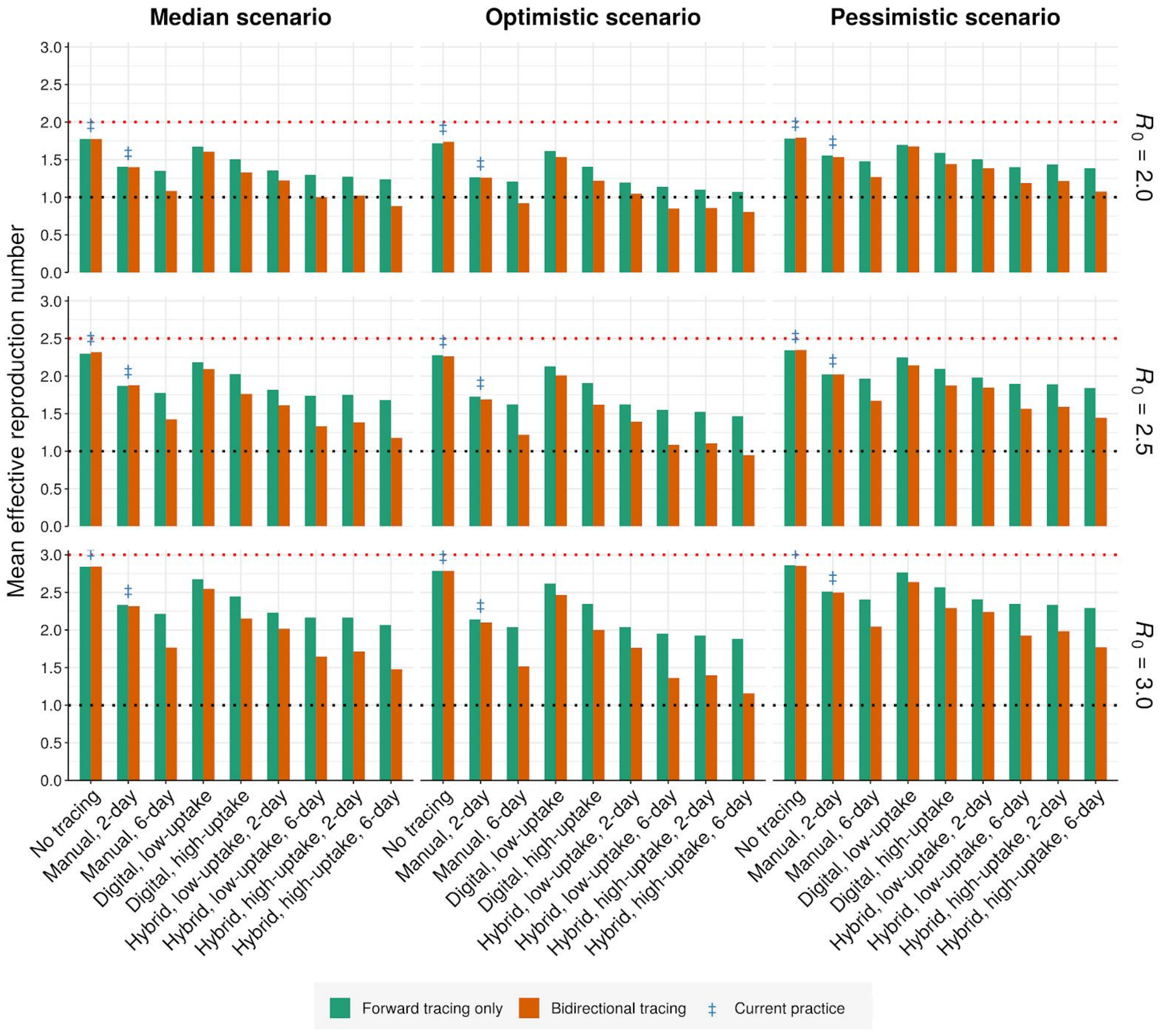

Supplementary Figure 32. Performance of different tracing strategies for controlling COVID-19 (no test required). As Supplementary Fig. 30, but assuming that tracing could be initiated from identified symptomatic cases without a positive test result. Mean effective reproduction number obtained under (left) median, (middle) optimistic, and (right) pessimistic scenarios (Supplementary Table 1), assuming a 90% baseline probability of trace success across 1000 runs. Blue double dagger symbols indicate conditions roughly corresponding to current practice in most regions. Low and high uptake correspond to 53% and 80% of cases, respectively, having chirp-enabled smartphones. Without tracing, forward and bidirectional are equivalent.

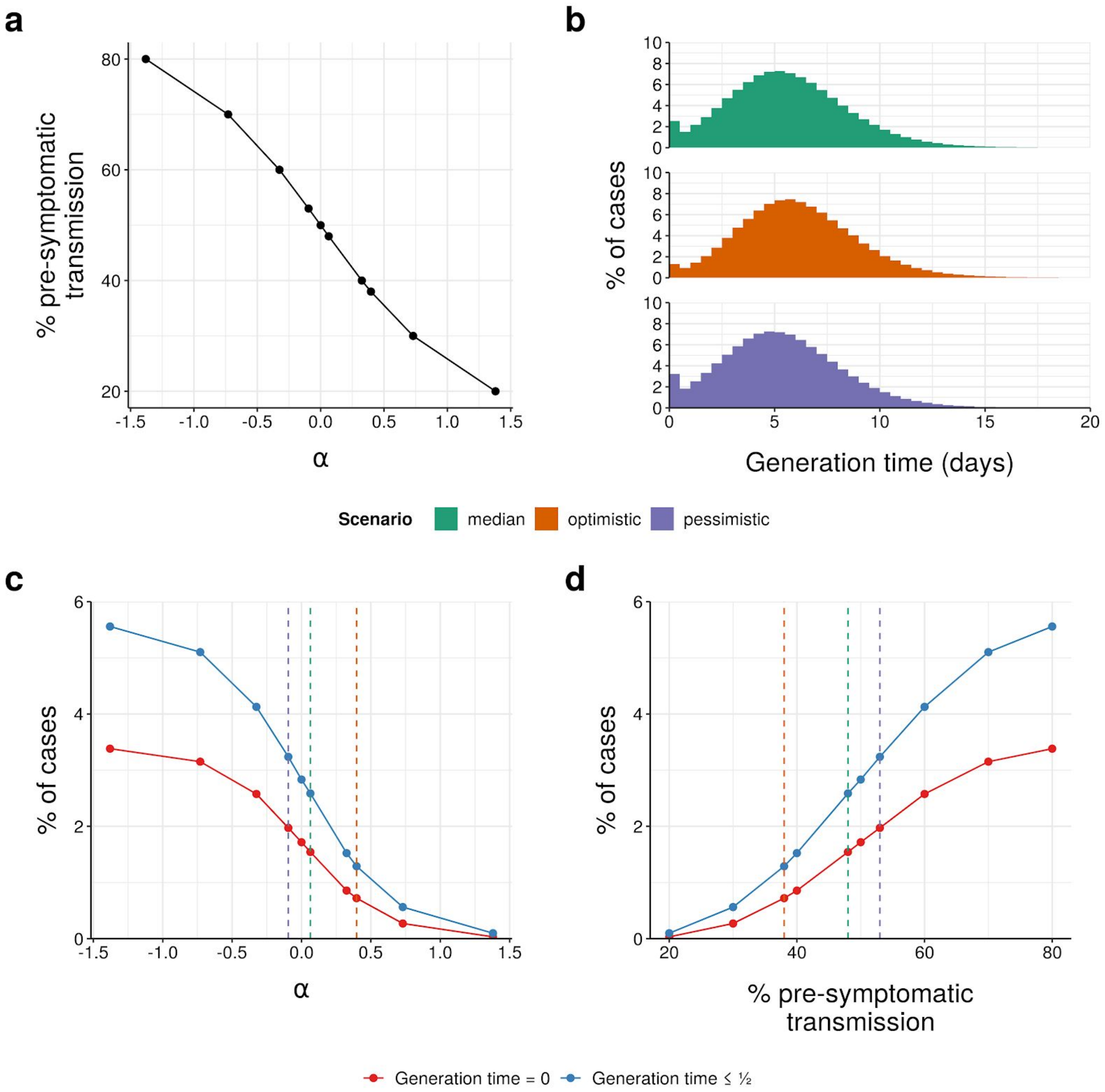

Supplementary Figure 33. Generation times and pre-symptomatic transmission. (a) Relationship between the skew (α) parameter of the skew-normal generation-time distribution and the fraction of pre-symptomatic transmission, given an SD parameter of 2. (b) Histograms of generation times for the median, optimistic and pessimistic scenario parameters. (c-d) Frequency of cases with zero (red line) or very small (blue line) generation times for (c) different values of the skew parameter or (d) the corresponding pre-symptomatic transmission frequencies. Vertical dashed lines correspond to the scenario of the corresponding colour from (b).
